# *Wars2* is a determinant of angiogenesis

**DOI:** 10.1038/ncomms12061

**Published:** 2016-07-08

**Authors:** Mao Wang, Patrick Sips, Ester Khin, Maxime Rotival, Ximing Sun, Rizwan Ahmed, Anissa Anindya Widjaja, Sebastian Schafer, Permeen Yusoff, Pervinder Kaur Choksi, Nicole Shi Jie Ko, Manvendra K. Singh, David Epstein, Yuguang Guan, Josef Houštěk, Tomas Mracek, Hana Nuskova, Brittney Mikell, Jessie Tan, Francesco Pesce, Frantisek Kolar, Leonardo Bottolo, Massimiliano Mancini, Norbert Hubner, Michal Pravenec, Enrico Petretto, Calum MacRae, Stuart A Cook

**Affiliations:** 1 Cardiovascular and Metabolic Disorders Program, Duke-NUS Medical School, National University of Singapore, 8 College Road, Singapore 169857, Singapore; 2 Department of Medicine, Yong Loo Lin School of Medicine, National University of Singapore, 1E Kent Ridge Road, Singapore 119228, Singapore; 3 Cardiovascular Division, Department of Medicine, Brigham and Women's Hospital and Harvard Medical School, 75 Francis Street, Boston, Massachusetts 02115, USA; 4 Medical Research Council Clinical Sciences Centre, Faculty of Medicine, Imperial College London, Hammersmith Hospital, Du Cane Road, London W12 ONN, UK; 5 National Heart Centre Singapore, 5 Hospital Drive, Singapore 169609, Singapore; 6 Institute of Physiology, Czech Academy of Sciences., 142 20 Prague 4, Czech Republic; 7 National Heart and Lung Institute, Royal Brompton Campus, Imperial College London, London SW3 6NP, UK; 8 Department of mathematics, South Kensington Campus, Imperial College London, London SW7 2AZ, UK; 9 Department of Radiological, Oncological and Anatomo-Pathological Sciences, University of Rome, 00161 Sapienza, Italy; 10 Cardiovascular and Metabolic Sciences, Max Delbrück Center for Molecular Medicine (MDC), 13125 Berlin, Germany; 11 Charité-Universitätsmedizin, 10117 Berlin, Germany; 12 DZHK (German Center for Cardiovascular Research), Partner Site Berlin, 13125 Berlin, Germany

## Abstract

Coronary flow (CF) measured *ex vivo* is largely determined by capillary density that reflects angiogenic vessel formation in the heart *in vivo*. Here we exploit this relationship and show that CF in the rat is influenced by a locus on rat chromosome 2 that is also associated with cardiac capillary density. Mitochondrial tryptophanyl-tRNA synthetase (*Wars2*), encoding an L53F protein variant within the ATP-binding motif, is prioritized as the candidate at the locus by integrating genomic data sets. WARS2(L53F) has low enzyme activity and inhibition of *WARS2* in endothelial cells reduces angiogenesis. In the zebrafish, inhibition of *wars2* results in trunk vessel deficiencies, disordered endocardial-myocardial contact and impaired heart function. Inhibition of *Wars2* in the rat causes cardiac angiogenesis defects and diminished cardiac capillary density. Our data demonstrate a pro-angiogenic function for *Wars2* both within and outside the heart that may have translational relevance given the association of *WARS2* with common human diseases.

## Abstract (web summary)

Blood supply to the heart is crucial for cardiac function. Here, the authors show that the mitochondrial tryptophanyl-tRNA synthetase, WARS2, drives blood vessel generation in zebrafish and rats and that inhibition of Wars2 diminishes blood vessel growth both within and outside in the heart, suggesting a new target for manipulating angiogenesis.

## Graphical abstract

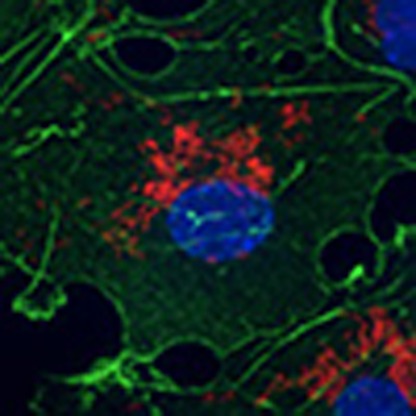

Angiogenesis describes the formation of new vessels from the existing vasculature^1^ and is a critical physiological process for matching oxygen and nutrient supply to tissue metabolic demands. In disease, pathological angiogenesis often occurs and this is an important process for cancers, retinal diseases and inflammatory conditions^2^,^3^. In the diseased heart, cardiac myocyte metabolic needs can exceed capillary vessel growth leading to diminished oxygen delivery and cardiac ischaemia^4^,^5^,^6^. While coronary flow (CF) is highly heritable^7^, large-scale genetic studies of CF in humans have not been possible due to the complexities and limitations of CF phenotyping^8^, although increasing CF remains an attractive target for therapeutic intervention^9^.

During angiogenesis, the angiogenic sprouting process requires the recruitment of tip and stalk cells from existing endothelium, followed by migration and proliferation of these cells in response to a chemoattractive tissue gradient. One of the most important pro-angiogenic signals is provided by vascular endothelial growth factor (VEGF), which activates quiescent endothelial cells (ECs) and promotes new vessel formation. The phenotypic fate determination of ECs into leading tip cells or supporting stalk cells depends on a highly regulated balance between VEGF-receptor and Notch-dependent signalling^10^, which remains incompletely understood. Cardiac vessels develop via angiogenic processes, although there is controversy regarding their EC origin^11^,^12^,^13^.

The limitations of effective ways to regulate angiogenesis relate, in part, to our incomplete understanding of the underlying biology that we postulated, may be improved through genetic studies of CF in the *ex vivo* rat heart, which provides a model for an indirect, quantitative measurement of capillary density and, by inference, angiogenesis.

In this work, we identify mitochondrial tryptophanyl-tRNA synthetase (*Wars2*) as a novel gene for cardiac capillary density and CF in the rat. The pro-angiogenic effect of *Wars2* is confirmed by loss- and gain-of-function approaches *in vitro* and in two *in vivo* models. Our findings describe a novel gene for angiogenesis both within and outside the heart.

## Results

### Determinants of CF in the rat

**Figure 1:**
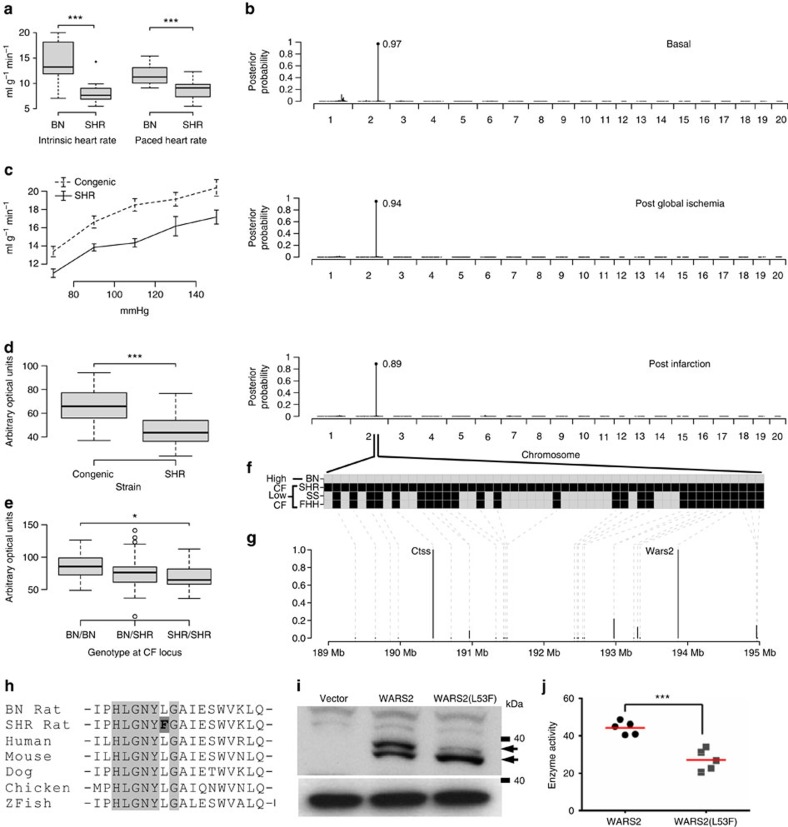
Mapping of coronary flow to the rat 2q34 locus and identification of *Wars2* as the candidate gene at the locus. (**a**) Coronary flow (CF) under intrinsic heart rate conditions in the Brown Norway (BN) and Spontaneously Hypertensive rat (SHR) (left panel, *n*=12) and under cardiac pacing conditions (right panel, *n*=20). (**b**) Genome-wide Evolutionary Stochastic Search (ESS) mapping^17^ of CF at three distinct experimental time points in an F2 cross (*n*=172) between the SHR and BN strains (*x* axis, rat autosomes; posterior probabilities for the peak SNP shown). (**c**) Replication of the CF QTL by congenic rescue in an SHR strain (SHR.BN2q34) encoding the BN genotype at the CF locus (*P*=3.2 × 10^−6^; *n*=6, SHR and 8, congenic; two-way analysis of variance (ANOVA) with Tukey's multiple comparisons test). (**d**) Capillary density (shown in arbitrary optical units) in the heart in SHR.BN2q34 strain (*n*=8) and the parental SHR (*n*=6) strain. (**e**) Capillary density (shown in arbitrary optical units) in F2 rats by BN/SHR genotype of the peak-associated SNP (*n*=10–16 per genotype). One-way ANOVA with Tukey's multiple comparisons test. (**f**) Refinement of the 2q34 locus using flanking markers to the QTL (Chromosome 2: 189.3 Mb and 194.5 Mb) and all protein damaging variation at the locus in SHR, Salt Sensitive (SS) or Fawn Hooded Hypertensive (FHH) rats (black squares; grey squares, no variation). (**g**) Candidate gene prioritization using BN, SHR, SS and FHH whole-genome sequences and PolyPhen2 prediction of protein damaging variants effect (y-axis) from (**f**). Grey dashed lines indicate physical position of genes. (**h**) Sequence alignments of Wars2(L53F) protein variation in the ‘HXGH motif'^19^ in the SHR, the BN rat and other species (zebrafish, ZFish). (**i**) Western blot of wild-type WARS2 and WARS2(L53F) protein. Arrows indicate WARS2- specific immunoreactive band (upper arrow, high molecular weight isoform; lower arrow, low molecular weight isoform); bottom panel, GAPDH immunoblot as loading control. (**j**) *In vitro* assay of canonical enzymatic activities (arbitrary optical units) of WARS2 and WARS2(L53F) (red line, mean). *t*-test: *P<0.05; ***P<0.001.

The rat is an excellent model of human cardiovascular physiology^14^,^15^ and CF has been determined in a large number of rat strains (http://pga.mcw.edu and^16^) with the Brown Norway (BN) rat having the highest CF of all rat strains (Supplementary Fig. 1). We took advantage of this knowledge and the fact that CF measurements provide an indirect but highly accurate and quantitative read out of capillary density to initiate genetic studies of CF in the rat. Using a controlled cardiac pacing protocol we confirmed the difference in CF between the BN rat and the Spontaneously Hypertensive Rat (SHR), which we have studied extensively^14^,^15^ (Fig. 1a). On the basis of these data, we hypothesized that CF in the rat is under genetic control as it is in humans^7^, and set out to dissect the genetic determinants underlying CF in a genetic intercross between the SHR (with low CF) and the BN rat (with high CF).

We generated a large F2 intercross (*n*=172) between BN and SHR strains and measured blood pressure (BP) *in vivo* followed by CF indexed to heart weight and left ventricular (LV) contractility and relaxation *ex vivo* in this population. CF occurs primarily during heart relaxation and, as expected, we observed that slower LV relaxation was associated with lower CF (*r*=−0.34, *P*=4.6 × 10^−6^, Pearson's correlation coefficient). There was no effect of BP on CF ruling out BP-related effects on CF in our experimental model (Supplementary Fig. 2).

### Mapping of CF to the rat genome

We carried out genome-wide genotyping of the F2 rats and used a multivariate Bayesian regression model that is an established tool for genome-wide genetic mapping of complex traits^17^ to identify the genetic control points of CF. Maximal CF (CF_max_) mapped to a genetic locus on rat chromosome 2q34 (posterior probability>0.89; single-nucleotide polymorphism (SNP) location: 191.7 Mbp; flanking markers: 189.3 Mbp and 194.5 Mbp) at all three experimental time points (Fig. 1b). The association of CF_max_ with the locus (+1.43 ml g^−1^ min^−1^ per BN allele, effect size ∼13%; Supplementary Fig. 3) was independent of BP, myocyte size or cardiac relaxation (Supplementary Figs 3 and 4) and was confirmed in a congenic rat strain (SHR.BN2q34; congenic interval 116.9–221.3 Mbp) (Fig. 1c). Two consomic strains, where the BN chromosome 2 has been introgressed to the Salt Sensitive (SS) or Fawn Hooded Hypertensive (FHH) backgrounds^16^, have higher CFs than respective parental strains further establishing a role for rat chromosome 2 on CF, which in the context of normal epicardial coronary arteries in the laboratory rat in the absence of neuro-humoral tone in the *ex vivo* heart would be expected to reflect cardiac capillary density.

### Wars2 regulates capillary density in the heart

Cardiac capillaries are small radius vessels present at variable densities in the heart that are important determinants of CF in the absence of arterial disease^4^,^5^,^8^, hence, we examined whether capillary density in the heart was influenced by the peak CF_max_ SNP. We observed reduced capillary density associated with the SHR allele (low CF) at the locus, an effect that was confirmed in the SHR.BN2 congenic (Fig. 1d,e). Taken together, these studies identify and replicate a discrete genetic locus for CF on rat 2q34 that also determines capillary density in the heart.

To identify protein coding variation at the CF locus with potential deleterious effects, we used whole-genome sequence data^18^ of the parental mapping strains (BN and SHR) and of the consomic strains (SS and FHH) (Fig. 1f,g). This prioritized two strongly protein damaging variants in low flow strains (SHR, SS and FHH) but not the high-flow strain (BN) within refined regions of the larger locus. These variants were encoded in *Cathepsin-S*, with variation at a partially conserved protein site (Supplementary Fig. 5) and a missense variant (L53F; common in rat strains (Supplementary Data 1)) in mitochondrial tryptophanyl-tRNA synthetase (*Wars2*, expressed in the heart (Supplementary Fig. 6)). The Wars2(L53F) variant occurred within a highly conserved ATP-binding ‘HXGH motif'^19^ that defines class I aminoacyl tRNA synthetases (ARSs) (Fig. 1h). During evolution many ARSs have acquired non-canonical functions, which can be enzyme activity independent^20^. It is established that while constitutively expressed, ARS loss-of-function can cause tissue-specific human disease^19^,^21^,^22^. Of potential relevance, the cytosolic tryptophanyl-tRNA synthetase (WARS) has been shown to influence angiogenesis^23^,^24^ although it is highly dissimilar to WARS2 (12.6% identity). Thus, we prioritized *Wars2* as the candidate gene for CF and capillary density at the locus based on its biological candidacy and the L53F mutation within its highly conserved ATP-binding site.

### Characterization of WARS2(L53F)

Both wild-type WARS2 and the WARS2(L53F) mutant localized to the mitochondria and were detected as two immunoreactive bands in whole-cell lysates (Fig. 1i and Supplementary Fig. 6). The WARS2(L53F) mutant exhibited a greater proportion of the lower molecular weight band, which reflected an increased localization of the mutant protein to the mitochondria (Supplementary Fig. 7). Given the L53F mutation lies within a highly conserved ATP-binding motif we tested WARS2 enzyme activity and observed a ∼40% reduction in activity of the WARS2(L53F) mutant (Fig. 1j and Supplementary Fig. 7). These data show that the WARS2(L53F) mutation affects post-translational processing of WARS2 and impairs its canonical enzymatic activity.

### *WARS2* regulates EC biology

**Figure 2:**
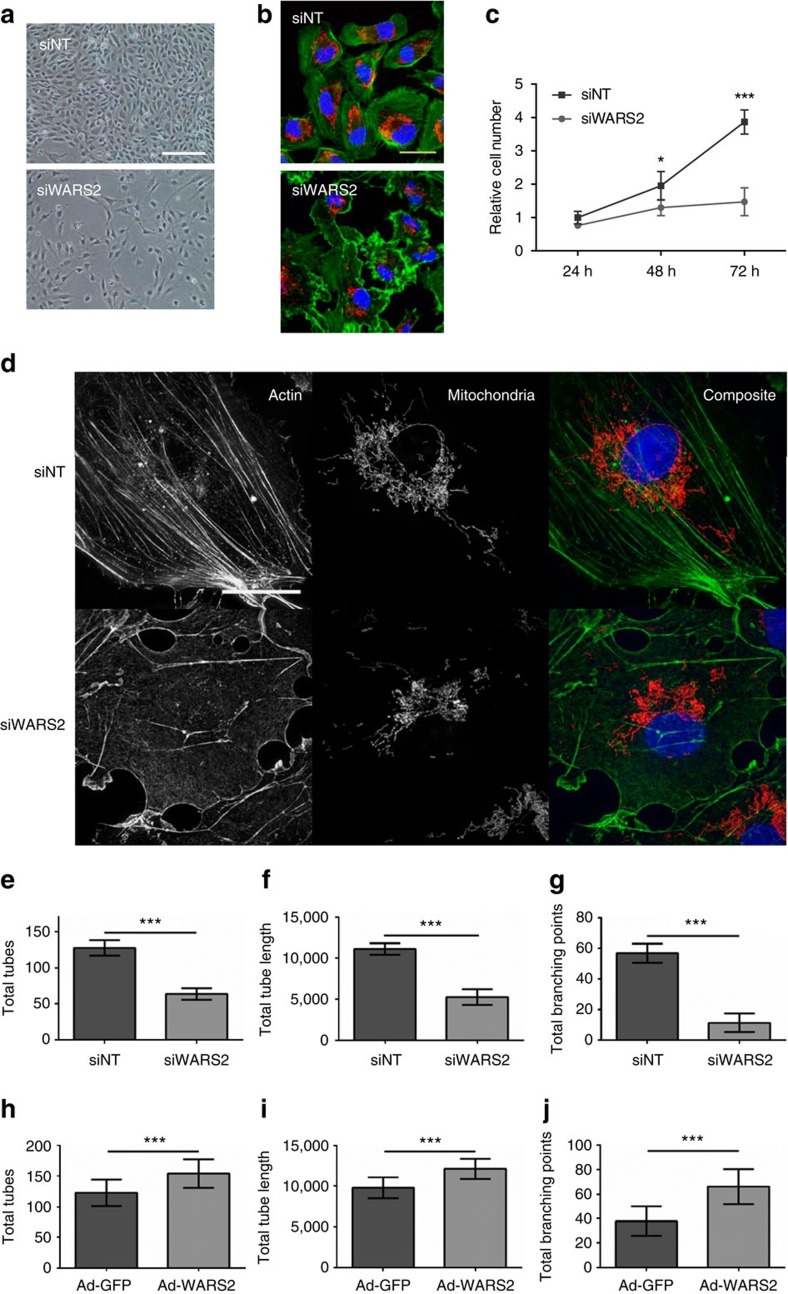
*WARS2* regulates endothelial cell morphology and angiogenic potential. (**a**) Bright field micrographs of endothelial cells (ECs) following transfection with either control siRNA (siNT) or siRNA against *WARS2* (siWARS2). Scale bar=200 μm. (**b**) Confocal microscopy of ECs transfected with siNT or siWARS2 (red, mitochondria; green, actin; blue, nucleus; scale bar =30 μm). (**c**) EC number in EC cultures transfected with siNT or siWARS2 (*n*=3 per condition, *t*-test). (**d**) Super-resolution microscopy of ECs transfected with siNT or siWARS2 and stained for actin (left), mitochondria (middle) and composite images with nuclear stain (right). Scale bar =25 μm. (**e–j**) Effects of *WARS2* in an *in vitro* model of EC angiogenesis. (**e**–**g**) *WARS2* loss of function; (**h–j**) *WARS2* gain-of-function. Total tubes (**e**, **h**), total tube length (in pixel) (**f**, **i**) and total branching points (**g**, **j**). *n*=8, *t*-test. **, *P*<0.01; ***, *P*<0.001.

Cardiac angiogenesis has an absolute requirement for EC proliferation, migration and capillary formation^4^,^5^, hence, we studied the effects of *WARS2* in ECs. Inhibition of *WARS2 in vitro* was associated with notable changes in EC morphology and a reduction in EC proliferative capacity (Fig. 2a–c). However, inhibition of other *AARS2* family members (HARS2 and LARS2) did not affect EC morphology or proliferative capacity showing gene-specific effects as opposed to gene family-generic effects (Supplementary Fig. 8). Confocal and super-resolution microscopy revealed that *WARS2* silencing caused EC spreading, abnormal membrane ruffling and a marked reduction in EC actin fibres that are centrally important for EC motility, division and polarity (Fig. 2b, d)^3^,^25^. We tested the effects of *WARS2* in an established *in vitro* model of angiogenesis: *WARS2* loss-of-function resulted in impaired angiogenesis (Fig. 2e-g) whereas *WARS2* gain-of-function enhanced angiogenesis (Fig. 2h-j).

### *Wars2* is a critical pro-angiogenic factor in zebrafish

**Figure 3:**
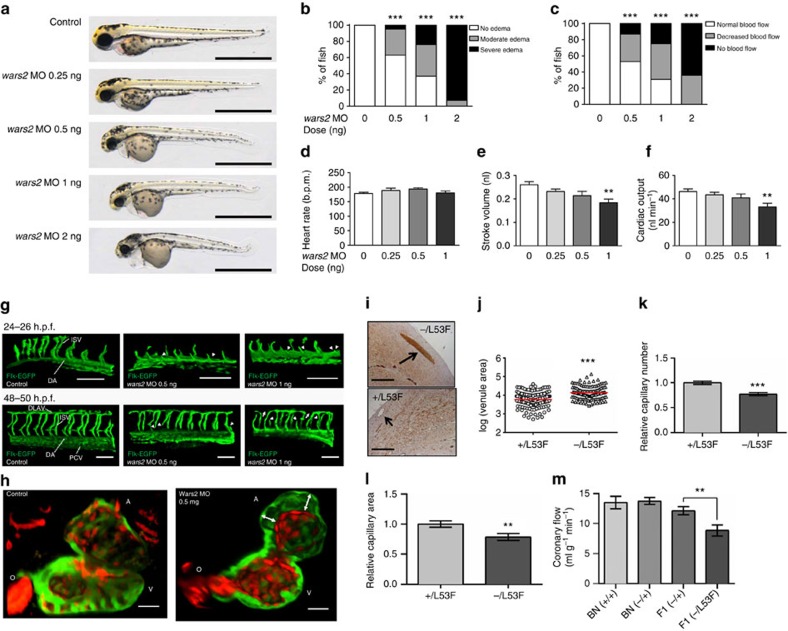
Effects of inhibition of *wars2* in the zebrafish and rat. (**a**) Zebrafish embryos injected with morpholinos (MO; 0.5 ng, 1 ng or 2 ng) against *wars2* or non-targeting MO (Control) and imaged at 72 h post fertilization (72 hpf). Scale bar=1 mm. (**b**, **c**) Percentage of fish with cardiac oedema (b) and normal, decreased or absent blood flow (c) at 72hpf. *n*=55, 62, 54, and 42 fish for doses of 0, 0.5, 1, and 2 ng *wars2* MO, respectively; Chi-square test. (**d**–**f**) Inhibition effect of *wars2* on heart rate (**d**), cardiac stroke volume (**e**) and cardiac output (**f**). *n*=15, 13, 16, and 15 fish for doses of 0, 0.25, 0.5, and 1 ng *wars2* MO, respectively; Dunnett's multiple comparison test after one-way analysis of variance (ANOVA). (**g**) 3D reconstruction of 2-photon Z-stack images of 24–26 hpf (top) and 48–50 hpf (bottom) *Tg(flk:EGFP)* transgenic zebrafish embryos. Green fluorescent trunk vessels are shown from controls and embryos with 0.5 or 1 ng of the *wars2* morpholino. Defects of intersegmental vessels (ISV, arrowheads) become obvious at 24 h.p.f.; disruptions of dorsal longitudinal anastomotic vessels (DLAV, arrows) occur at 48 hpf Fish anterior end is located to the left in all images. DA: dorsal aorta; PCV: posterior cardinal vein. Scale bar=100 μm. (**h**) Optical slice through 3D-reconstructed images of hearts of zebrafish embryos 5 days post fertilization using *in vivo* 2-photon microscopy. *Tg(myl7:GFP;flk:dsRed)* zebrafish, showing green fluorescence in myocardium and red fluorescence in endocardium, with separation (arrows) of cell layers after *wars2* knockdown (a: atrium, o: outflow tract, v: ventricle.). Scale bar, 30 μm. (**i**–**m**), Effects of *Wars2* loss-of-function on capillary density and coronary flow in rat heart. (**i**) Histological section of rat hearts stained for CD31 showing very large sub-epicardial veins in F1(*Wars2*^−/L53F^) rats but not F1(*Wars2*^+/L53F^) rats. Scale bar=500μm. (**j**) Sub-epicardial venule areas (log scale, arbitrary units) in F1 rats (*n*=5, red line, mean). (**k**) Relative capillary density in F1 rats (*n*=5). (**l**) Relative capillary area in the heart F1 rats (*n*=5). (**m**) *Ex vivo* quantification of coronary flow under paced conditions in wild-type BN(*Wars2*^+/+^), *n*=9; BN(*Wars2*^−/+^), *n*=7; F1(*Wars2*^+/L53F^), *n*=7 and F1(*Wars2*^−/L53F^), *n*=11. **j–i**, *t*-test; m, one-way ANOVA with Tukey's multiple comparisons test. **P<0.01; ***P<0.001.

To test the effects of *wars2* on cardiac angiogenesis *in vivo* we first used the zebrafish (ZF), a preferred model system for studying vascular biology with well-developed transgenic lines and optical imaging techniques for the analysis of EC biology^26^. Morpholino-mediated *wars2* knockdown (titrated to effect; Supplementary Fig. 9) was associated with pericardial oedema and early ZF death (Fig. 3a–c and Supplementary Fig. 10), which could be rescued by transgenic *hsaWARS2* expression (Supplementary Fig. 11). *Wars2* inhibition caused marked cardiac contractile failure (Fig. 3d–f) as seen previously following the inhibition of *vegfa*^27^ or *ve-cadherin*^28^, prototypical determinants of angiogenesis.

There was a marked defect in the patterning of intersegmental vessels (ISVs) throughout the trunk of zebrafish embryos and in dorsal longitudinal anastomotic vessels (DLAVs) after *wars2* inhibition showing a direct effect of *wars2* on angiogenesis outside the heart (Fig. 3g and Supplementary Fig. 12). Time-lapse recordings of Tg(flk:EGFP) embryos starting at 20 h post fertilization showed that *wars2* knockdown leads to delayed, or in some cases absent, elongation of ISVs and missing connections of DLAV segments (Supplementary Movies 1, 2, 3, 4, 5, 6). This observation following *wars2* inhibition is similar to previous findings of incomplete growth of multiple ISVs during early development after disruption of *vegfa*, *vegfc*, the *vegfr-2* orthologs *kdra* and *kdrb* or the *vegfr-3* ortholog *flt4* (ref. 29), suggestive of interaction of *wars2* with these genes or their downstream pathways.

Using myocardial- and endothelial-specific fluorescence protein-expressing ZF lines, we studied the heart phenotype in greater detail and observed that *wars2* knockdown led to pathological separation of the EC endocardial layer from the myocardial cell layer (Fig. 3h; Supplementary Fig. 13 and Supplementary Movies 7,8), as seen previously following inhibition of *ve-cadherin*^28^. There was markedly less infiltration of ECs in myocardial trabeculae in the ventricle after *wars2* knockdown (Supplementary Fig. 14). The endocardium is a major source of ECs for the development of the coronary vasculature in zebrafish^30^ and also important in mammals^31^. Taken together, these data demonstrate that disrupted *wars2* function leads to impaired angiogenesis outside the heart and disrupted endocardial to myocardial apposition and interaction within the heart, reminiscent of *vegfa* effects^27^,^29^.

### *Wars2* inhibition in the rat causes cardiac angiogenesis defects

To investigate the effects of *Wars2* in a mammalian model, we generated a *Wars2* targeted rat on the BN (wild-type *Wars2*) background using zinc finger nucleases (ZFN) (Supplementary Fig. 15). Heterozygote (*Wars2*^−/+^) rats were born in usual Mendelian ratios and appeared normal, in keeping with the recessive nature of *ARS2* mutations in humans and their counterparts in mice^19^,^21^,^22^. Homozygous deletion (*Wars2*^−/−^) was embryonic lethal (<E8.5), which has also been documented in the uncharacterized *Wars2*^−/−^ mouse (www.jax.org). In an attempt to avoid embryonic lethality, we crossed BN (*Wars2*^−/+^) to SHR (*Wars2*^L53F/L53F^) to generate F1 animals that were genetically identical apart from the *Wars2* locus: F1(*Wars2*^+/L53F^) or F1(*Wars2*^−/L53F^), respectively. Given the loss-of-function associated with the L53F allele we reasoned that the F1 (*Wars2*^−/L53F^) would represent a compound hypomorph as compared with the F1(*Wars2*^+/L53F^).

Histological analyses of the heart revealed very large sub-epicardial veins in the F1(*Wars2*^−/L53F^) as compared with F1(*Wars2*^+/L53F^) (Fig. 3i,j), a phenomenon previously observed in the *Vegfa*-deleted mouse heart^11^. F1(*Wars2*^−/L53F^) rats had fewer and smaller capillary vessels than the F1(*Wars2*^+/L53F^) controls (Fig. 3k and l and Supplementary Fig. 16), in keeping with our data from the F2 mapping and congenic strains (Fig. 1). Our foundational genetic experiments in the rat identified a CF_max_ QTL at the rat 2q34 locus, hence we examined CF_max_ in F1 (*Wars2*^+/L53F^) and F1(*Wars2*^−/L53F^) rats. As compared with the F1(*Wars2*^+/L53F^) rats, the F1(*Wars2*^−/L53F^) rats had significantly lower CF_max_ (Fig. 3m) in the absence of cardiac dysfunction (Supplementary Table. 1). These data confirm that *Wars2* loss-of-function is causally related to diminished cardiac angiogenesis and reduced CF.

### Inhibition of *WARS2* impairs EC proliferation

**Figure 4:**
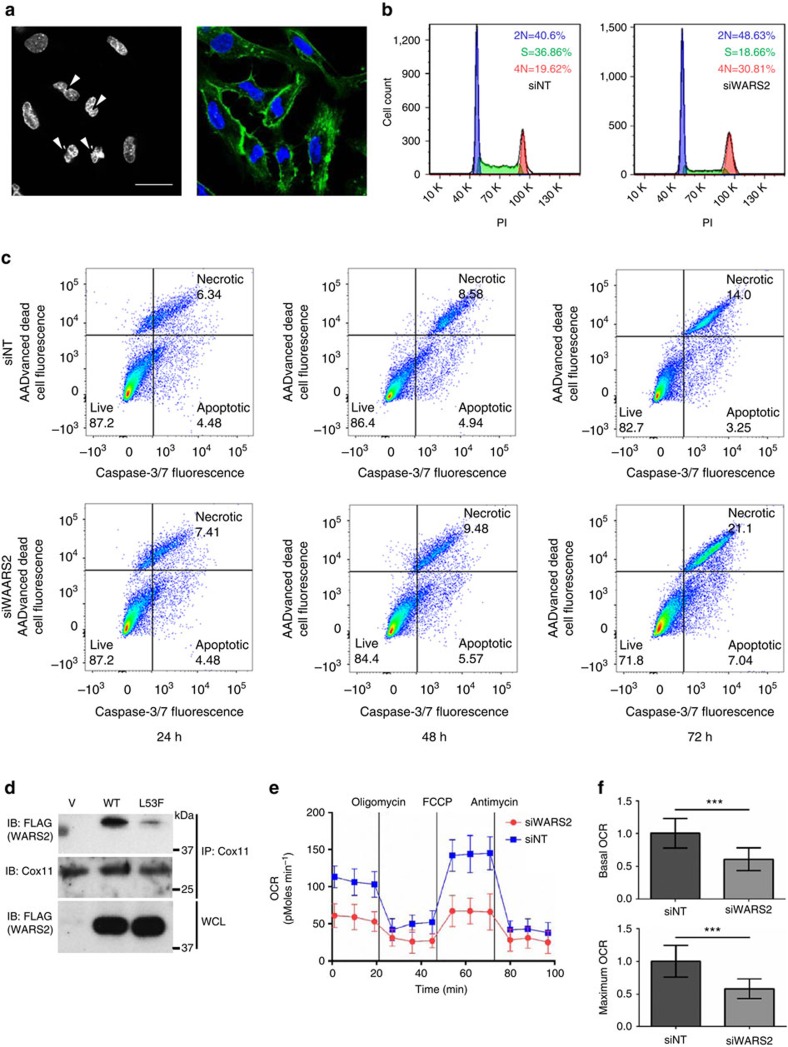
Effects of *WARS2* on endothelial cell viability and oxygen consumption. (**a**) SiRNA-mediated inhibition of *WARS2* in endothelial cells (ECs) results in multiple cells with two or more incompletely separated nuclei. Left panel, nuclei (arrow heads); right panel, nuclei (blue) and actin (green). Scale bar=50 μm. (**b**) FACS analysis of the cell cycle in proliferating ECs treated with non-targeting siRNA (siNT) or siRNA against *WARS2* (*siWARS2*) shows a reduction in ECs in the S phase and an increased number in the G2/M phase with siW2. The experiment was repeated (*n*=3) with similar results (**c**) FACS analysis of caspase-3/7 activation and cell death over a 72-h time course. ECs were transfected with non-targeting siRNA (siNT) or siRNA against *WARS2* (siWARS2). The experiment was repeated with similar results (and see Supplementary Fig. 14). (**d**) Immunoblot (IB) of COX11 and Flag in Cox11 immunoprecipitates (IP: COX11) and of whole cell lysates (WCL) from cells expressing either vector alone (V), Flag-tagged wild-type WARS2 (WT) or Flag-tagged mutant WARS2(L53F) (L53F). (**e**) Oxygen consumption rates (OCRs) in ECs transfected with non-targeting siRNA (siNT, blue boxes) or siRNA against *WARS2* (siWARS2, red circles). A representative experiment is shown; the experiment repeated 5 times with similar results. (**f**) Quantification of basal (upper panel) and maximal (lower panel) OCRs. *n*=3, *t*-test. **, *P*<0.01. ***, *P*<0.001.

To begin to explore the cellular mechanisms underlying *WARS2* effect in ECs, we used *in vitro* approaches to test whether the reduction in EC number observed following *WARS2* inhibition (Fig. 2c) was due to impaired EC proliferation and/or increased EC death. Following *WARS2* inhibition, ECs frequently contained incompletely separated nuclei (Fig. 4a) and this was associated with an early increased number of ECs in the G2/M phase of the cell cycle (Fig. 4b), consistent with EC cell cycle arrest and incomplete cytokinesis^32^. We then examined cell death and observed that inhibition of *WARS2* promoted EC cell death at later time points (Fig. 4c and Supplementary Fig. 17). Overall these data show that an effect of *WARS2* inhibition is on EC cell cycle arrest in G2/M, perhaps related to impaired cytokinesis, followed by later onset activation of pro-apoptotic pathways and cell death.

### Inhibition of *WARS2* reduces mitochondrial oxygen consumption

For molecular studies, we began by performing unbiased *WARS2* gene-centric co-expression analysis using a large (*n*=128) human heart RNA sequencing data set (Accession Number: PRJEB8360). This revealed that *WARS2* gene expression was most highly positively correlated with *COX15* (r=0.57, *P*=3.2 × 10^−08^, Pearson's correlation coefficient) and *COX11* (*r*=0.55, *P*=4.2 × 10^−07^, Pearson's correlation coefficient) (Supplementary Data 2). The observed co-expression, and inferred interaction, between WARS2 and COX11 was substantiated in separate, genome-wide yeast two-hybrid experiments that identified a single WARS2-interacting protein: COX11. We confirmed the WARS2/COX11 interaction by co-immunoprecipitation and detected that this interaction was diminished for the WARS2(L53F) mutant (Fig. 4d). Given the canonical role of *WARS2*, we tested the effect of *WARS2* inhibition on mitochondrial respiration in ECs. Following *WARS2* knockdown there was a reduction in oxygen consumption rates (Fig. 4e,f), which would be expected to inhibit proliferating ECs that have a low respiratory reserve during angiogenesis^33^. However, it remains to be ascertained whether mitochondrial dysfunction is the major determinant of *WARS2* effect on angiogenesis or whether other non-canonical functions are at play.

## Discussion

Here we identify *Wars2* as a new gene for angiogenesis both within and outside the heart. In human genome-wide association studies, heritable *WARS2* gene expression was identified in breast cancers^34^ and the *WARS2* locus was associated with cardio-metabolic phenotypes^35^ that were also linked to the *VEGFA* locus^35^. Intriguingly, genetic studies in the mouse implicated *Wars2* in capillary formation in the skin^36^. The WARS2(L53F) variant we identified is associated with reduced enzymatic activity, which can unveil non-canonical effects of ARSs that are often, perhaps surprisingly, tissue specific. We note that, in particular in the zebrafish, there are several mutations in other *ARS* genes that cause vascular defects. Interestingly, deficiency in several ARS genes (*iars*, *sars* and *tars*) lead to increased branching of ISVs, showing that *ARS* genes can have both pro- and anti-angiogenic effects^37^,^38^,^39^,^40^. On the other hand, other zebrafish models with profoundly impaired mitochondrial function do not show the cardiovascular phenotypes^41^,^42^,^43^ that we observed (Fig. 3a-h) indicating that the effects of *wars2* disruption may be not due to a generic effect of mitochondrial dysfunction.

The effects of *wars2* knockdown in zebrafish embryos are similar to what has been observed previously in mutants with disrupted *VEGF* function^29^. More specifically, the defects in angiogenic sprouting of ISVs, likely due to a lack of migration and/or proliferation of tip and stalk cells, respectively, suggest that *WARS2* might play a role in the signalling mechanisms downstream of the *VEGFR* responsible for tip and stalk cell behaviour^44^, potentially involving the balance between *VEGF*- and *Notch*-dependent signalling. *VEGF* is known to be a major regulator of cardiac angiogenesis^45^, and deletion of either myocardial *Vegf-a* or endocardial *Vegfr-2* has been shown to prevent normal coronary vasculature development in mice^11^. Interestingly, disruption of *VE-cadherin*, an endothelial-specific adhesion molecule mediating downstream effects of *Vegf-a (vegf-a)*, prevented sprouting angiogenesis in mice^46^ and led to a separation between the myocardium and endocardium in zebrafish embryos^28^. A recent study also showed that *Notch1* activation promotes the tight interaction between endocardium and myocardium, leading to cardiac trabeculation via *ephrin B2* and *neuregulin* activation^47^. Our comparable observations of decreased cardiac capillary density in *Wars2*(^−/L53F^) rats as well as the myocardial–endocardial separation after *wars2* knockdown in zebrafish support a hypothesis that *WARS2* interacts with pathways downstream of *VEGF* and *Notch*. Overall, our data show that *WARS2* is a novel determinant of angiogenesis in the heart and other tissues perhaps acting as an integrator of pro-angiogenic signalling, directing cell motility and division to enable EC migration and proliferation.

## Methods

### Publically available CF data

Previously derived CF data from rat strains was obtained from Physgen via the web resource (http://pga.mcw.edu/)

### Parental strains and BN X SHR F2 population

BN and SHR strains were used to validate publically available data. Rats were bred by a monogamous mating system. BN females were crossed with SHR males to produce BN X SHR (BXH) F1 animals and a reciprocal cross was performed to obtain SHR X BN (HXB) animals. F1 BXH animals were intercrossed to generate F2 BXH animals and F1 HXB animals were intercrossed to generate F2 HXB animals. Animals were maintained at the Central Biomedical Services facility, Imperial College, London, and housed at a maximum of five rats per cage. The animals had ad libitum access to standard rat chow and sterile water. Except for breeding, animals were separated according to sex. Rats were maintained on a twelve-hour diurnal cycles by automatic light switching. All procedures were performed in accordance with the UK Animals (Scientific Procedures) Act of 1986. All rats for baseline and mapping studies were purchased from Charles River UK Limited (Margate, UK). Male rats aged 10–16 weeks were used throughout.

### Blood pressure

BP was measured by cannulation of the carotid artery before cardiac excision. An ultra-miniature 2 mm pressure catheter (MPVS-Ultra Single Segment Foundation System, ADI instruments) was used. Animals were anaesthetized using inhaled 4% Isoflurane. Data were captured using LabchartPro software (ADI instruments). After carotid cannulation, the concentration of inhaled Isoflurane was reduced from 4% to 1.5% to eliminate BP lowering effects of high Isoflurane concentrations.

### Coronary flow

CF was measured using a Langendorff preparation. Following excision, the heart was placed in ice-cold Kreb's buffer and transferred to apparatus. The aorta was secured using 3/0 silk. The heart was perfused with a modified Krebs–Henseleit buffer solution^48^ at 37 °C in a jacketed reservoir and continuously gassed with carbogen solution (95% Oxygen, 5% CO2). The left atrium (LA) was removed and a fluid-filled latex balloon was placed in the left ventricular (LV) cavity. LV contractility (LV dP/dt_max_) and LV relaxation (dP/dt_min_) were derived from LV pressure. Hearts were paced at 360 beats per minute (b.p.m.). The perfusate was maintained at constant pressure (90 mm Hg). CF was measured by an inline ultrasonic flowmeter. Hemodynamic data was captured continuously by LabchartPro software (ADI instruments). The isolated heart preparation was studied at baseline for 15 min, following one minute of global ischaemia and during reperfusion following ligation of the proximal left anterior descending artery for 35 min.

### Genotype data

DNA from F2 rats was extracted from tail tissue. High-throughput genotyping was performed using Illumina's GoldenGate assay on a custom genotyping beadchip. 16,543 SHR SNPs reported by Consortium *et al*.^49^ and the surrounding 160 bp sequence for each SNP were retrieved based on SHR genome sequence^50^. SNPs and their surrounding sequence were submitted to Illumina for bioinformatic assessment, and attributed a quality score. 768 high-quality SNPs that were uniformly distributed throughout the genome were selected for the custom beadchip. Following hybridization and imaging, genotypes were called using the GenomeStudio software, and the GenCall algorithm^51^. Parental and F1 samples were added to the F2 samples as controls of genotyping quality. SNPs with low mean normalized intensity (*R*<0.2), low MAF (MAF<0.1) or deviation from Hardy–Weinberg equilibrium were excluded. SNPs were also manually curated. The overall call rate was of 93%. Missing genotypes were imputed using fastPhase. Following all QC steps, 172 genotypes were sued for mapping experiments.

### CF mapping

CF QTLs were mapped to the rat genome using a Matlab implementation of ESS^17^, an established tool for genome-wide genetic mapping of complex traits. This is based on a fully Bayesian 'variable selection' strategy that is aimed to identify the best set of predictors using a linear regression framework, which, here has been used to identify genome-wide the best set of SNPs (‘predictors') that predict variation in CF (‘phenotype'), which have measured in the F2 cross between the SHR and BN strains. Six CF phenotypes (mean and maximum; at the three experimental time points) were included in the model as dependent variables and SNP genotypes were used as regressors in the model. To increase robustness to outliers, fixed effect for each individual were added to the regressors and included in the variable selection process. Default parameters were used for ESS. A marginal posterior probability of 0.8 was required to identify genetic regulation of the phenotype by a SNP. The corresponding haplotype was obtained, by taking the range between the two markers surrounding the marker selected by ESS.

### Prioritization of genes at the locus using genomic sequences

Deleterious potential of non-synonymous variants was assessed using polyphen 2 prediction^52^ for all genes at the locus in the BN, SHR, FHH and SS rats using whole-genome sequence data^18^. Variants with a predicted probability of being deleterious over 0.5 were considered as potentially damaging.

### SHR.BN chromosome 2 congenic strain

The SHR.BN-(D2Rat171/D2Arb24) congenic strain was derived by selective backcross breeding to the progenitor SHR strain (SHR/Ola) by transferring a differential segment of chromosome 2 from a normotensive BN (BN/Crl) strain (Charles River Laboratories, Wilmington, Massachusetts, USA). After 10 generations of selective backcrossing to the SHR progenitor strain, the differential segment was fixed by intercrossing heterozygotes and selecting for offspring inheriting the homozygous BN chromosome segment^53^. Rats were housed in an air-conditioned animal facility and allowed free access to diet and water. Male rats were used (aged 8–16 weeks) All experiments were performed in agreement with the Animal Protection Law of the Czech Republic and were approved by the Ethics Committee of the Institute of Physiology, Czech Academy of Sciences, Prague.

### CF studies of congenic rats

Animals were anaesthetized with intraperitoneal injection of thiopental, hearts were rapidly excised and perfused according to Langendorff under initial constant pressure of 70 mm Hg with non-recirculating modified Krebs–Henseleit solution^48^ gassed with 95% O_2_ and 5% CO_2_ (pH 7.4) and maintained at 37 °C. After 10-min perfusion at spontaneous heart rate a co-axial bipolar electrode was placed on the right atrium and pacing was commenced at 360 b.p.m. Five minutes later the perfusion pressure was increased stepwise up to 150 mm Hg. CF was measured after 5-min stabilization at each step by timed collection of coronary effluent and then normalized to heart weight.

### Rat cardiac capillary and vein histo-morphometry

After fixation (4% Formaldehyde), short-axis heart slices were processed (*n*=5 for each group) for paraffin embedding. Multiple 4-μm-thick sections were de-paraffinized, rehydrated and stained with hematoxylin and eosin for microscopic evaluation of morphology. To assess capillary density immune-histochemistry examination was performed using mouse anti-RECA1 antibody (1:500, ab22492, Abcam). All the animals were examined by a dedicated pathologist, blinded to genotype. Mid ventricular myocardial region was selected to measure capillary density in each heart for every single genotype. Particle thresholding was manually determined for each heart to avoid staining intensity bias that could arise from different staining batch. Briefly background subtraction was applied using the ‘rolling ball radius' algorithm to remove light inconsistency of the background. Particle thresholding was afterwards determined using default method on ImageJ, after splitting red, green and blue (RGB) channels and selecting the green channels (which provide better contrast on peroxidase staining) to avoid any non-capillaries specific thresholding. Cardiac myocyte size was determined by planimetry. Assessment of capillary density was performed using ImageJ software (NIH): briefly colour images of 10 random fields at × 40 original magnification were taken, avoiding intramural arterioles, fibrosis and other potentially biased structure, colour thresholding was used to identify capillaries and automated particle analysis was performed. Sub-epicardial venules were planimetered. Data are reported as arbitrary optical units or relative to control levels.

### Production of recombinant WARS protein

WARS2 wild-type and WARS2(L53F) mutant proteins were prepared and purified by Genscript using the Baculovirus expression system. Recombinant WARS was prepared in house using an overnight Rosetta (Novagen) culture transformed with pEX-N-GST-WARS diluted in 1 L Luria-Bertani broth, shaking at 180 r.p.m. at 37 °C. Protein expression was induced with 1 mM IPTG and incubated at 16 °C for 16 h. The induced culture was harvested by centrifugation at 5,000*g* for 15 min, followed by resuspension in lysis buffer (50 mM Tris pH 7.5, 150 mM NaCl, 1% sodium deoxycholate, 1% triton, 25% glycerol, 1 mg ml^−1^ lysozyme, 1 mM DTT and complete protease inhibitor cocktail (Roche)) followed with sonication. The lysate was cleared by centrifugation and the supernatant mixed with 2 ml of glutathione sepharose 4B resin (GE Healthcare) and resuspended in buffer (50 mM Tris pH 7.5, 150 mM NaCl, 25% glycerol, 1 mM DTT). The mixture was then applied to a glass econo-column (BioRad) and resin-bound WARS washed and then eluted in fractions of 2 ml elution buffer (50 mM Tris pH 8.0, 150 mM NaCl, 25% glycerol, 20 mM of L-glutathione reduced (Sigma) and 1 mM DTT). Fractions were run on SDS–PAGE gel and stained with Coomassie blue to check for purity and pure fractions pooled and buffer exchanged with 10 mM HEPES pH 7.5, 150 mM NaCl, 10% glycerol and 0.005% P20.

### Alkaline Phosphatase Treatment (Bandshift assay)

Cell lysates of HEK293 cells (CRL-1573, ATCC) transfected with FLAG-*SPRY2*, FLAG-*WARS2*, FLAG-*WARS2(L53F)* or the empty vector control were harvested in lysis buffer without sodium orthovanadate and subjected to incubation with calf intestinal phosphatase (CIP, New England Biolabs) for 2 h at 37 °C using bovine serum albumin as control.

### siRNA and adenovirus

Small interfering RNA (siRNA) targeting WARS2 (5′-CCGACAUUCUGUUGUACAAdTdT-3′, 5′-UUGUACAACAGAAUGUCGGdTdT-3′), HARS2 (5′-CCAACUGAAAGCACAUCAAdTdT-3′, 5′-UUGAUGUGCUUUCAGUUGGdTdT-3′), LARS2 (5′-CCACAAAGUUGGACACAAAdTdT-3′, 5′-UUUGUGUCCAACUUUGUGGdTdT-3′) and non-targeting control (SN001-10D) were purchased from SABio. WARS2 adenovirus (SL179531) and GFP adenovirus (SL100708) were purchased from SignaGen Laboratories.

### Cell culture and genetic manipulation

HEK293 cells were purchased from ATCC (CRL-1573). They were cultured and maintained in DMEM containing 10% fetal bovine serum (FBS), 6 mM L-Glutamine, 100 U ml^−1^ Penicillin and 100 μg ml^−1^ Streptomycin in a humidified incubator with 5% CO_2_. Cells were transfected using Lipofectamine 2000 (Invitrogen) according to the manufacturer's instructions. Human vein vascular ECs (HUVECs) were purchased from Lonza (C2519A) and were cultured with EGM-2 BulletKit medium (Lonza) in a humidified incubator with 5% CO_2_. HUVECs (P3 to P4) were harvested at confluence of 80–90% and subcultured into six-well plate (Falcon), μ-Slide 8 Well (ibidi) or 100 mm petri-dish (Falcon) at the density of 5,000 cells per cm^2^. *WARS2* siRNA and non-targeting control siRNA were transfected into HUVECs by using lipofectamine RNAiMAX transfection reagent (Invitrogen) according to the manufacturer's instruction. Medium was replaced with fresh EGM-2 BulletKit medium after 24 h. At ∼50% confluence, HUVECs were changed into low serum medium (EBM-2 supplemented with 1% FBS, Lonza). Adenovirus expressing human *WARS2* or GFP was added at a multiplicity of infection of 100.

### Yeast two-hybrid screening

Screening was performed by Hybrigenics Services SAS (Paris, France) using WARS2 (aa 20-360; N-LexA-WARS2-C fusion) against human ventricle and embryo heart library.

### Cellular phenotyping and microscopy

White field images were acquired by Eclipse TS100 inverted microscope (Nikon) with DS-Fi2 camera (Nikon). Images were acquired 72 h after transfection. Adherent HUVECs were harvested by trypsinization and counted using an automated cell counter (Countess Automated Cell Counter; Invitrogen).

### Tube formation assay

After 48 h siRNA transfection or 48 h adenovirus transduction, HUVECs were harvested and seeded in 96-well plates, pre-coated with 50 μl matrigel (Corning Matrigel matrix, growth factor reduced) per well, at the density of 8,000 cells per well. After culturing in full EGM-2 medium for 8 h, images were acquired for each well (at central position) at × 5 objective (DM3000 inverted microscope, Leica). Images were analysed by WimTube (Wimasis Image Analysis).

### Cell cycle analysis by flow cytometry

HUVECs were harvested and counted. Cells were washed in PBS and fixed in 70% cold ethanol while vortexing, stained using × 1 Propidium Iodide/RNase Staining Solution (Cell Signalling Technology) and analysed by FACS (BD LSR II flow cytometer system, BD Biosciences). Cells were gated and analysed by FlowJo software.

### Cell death analysis by flow cytometry

HUVECs were collected and counted. Cell were stained with CellEvent Caspase-3/7 green flow cytometry assay kit (Molecular Probes) according to manufacturer's instruction. Cells were then analysed by FACS (BD LSR II or FACSARIA III system; BD Biosciences). Cells were gated and analysed by FlowJo software. In separate experiments, HUVECs were harvested at 48 h and labelled with Image-iT DEAD Green viability stain (Molecular Probes) and subjected to FACS analysis as above.

### Seahorse assay of mitochondrial function

After 48 h of siRNA transfection, HUVECs were harvested and seeded into XF cell culture microplate (XF24 FluxPak, Seahorse Biosciences) at the density of 40,000 cells per well. Oxygen consumption rate was then recorded by using XF24 extracellular flux analyser (Seahorse Biosciences). Drugs sequentially: oligomycin (1 μM), FCCP (3 μM) and antimycin (2.5 μM).

### Confocal and super-resolution microscopy

HUVECs cultured in 8 well chamber slides were fixed with 4% formaldehyde. Cells were permeabilized with 0.1% Triton X100 and incubated with Alexa Fluor 488 phalloidin (Molecular Probes, 1:200 in PBS, 30 min). Cell nuclei were stained with DAPI (Molecular Probes, 1:1,000 in PBS, 5 min) and cells mounted with ProLong gold antifade mountant (Molecular Probes) or VECTASHIELD mounting medium (Vector Laboratories). Images were acquired using a confocal laser scanning microscope (LSM 710, Zeiss) and super-resolution structured illumination microscope (ELYRA PS.1, Zeiss). Super-resolution images were post processed by ZEN software (Zeiss) with SIM module according to manufacturer's instruction. Scale bar was added to all images by ImageJ software.

### Immunostaining

After permeablization with 0.1% Triton X-100 for 30 min, cells were washed three times by PBS and blocked in 1% bovine serum albumin for 30 min. Primary antibody (mouse anti-COX4, 1:1,000, Abcam) was then applied and kept overnight at 4 °C, followed by washing three times in PBS and staining with secondary antibody (goat anti-mouse Alexa Fluor-555, 1:1,000, Molecular Probes) for 30 min at room temperature. Samples were washed three times again in PBS and mounted in ProLong gold antifade. Finally, samples were stored in humidified chamber and stored in 4 °C until imaging.

### RNA extraction and quantitative PCR studies of cell samples

For tissues: 50 mg of tissue was homogenized in 1 ml TRIzol Reagent (Invitrogen) with homogenizing beads using MagNA Lyser (Roche) at 6,000 r.p.m. for 20 to 30 s, following 5 min incubation at room temperature. For cultured cells: 1 ml TRIzol reagent was directly applied to the cells in the culture dish per 10 cm^2^. Chloroform was then added (0.2 ml per 1 ml of TRIzol Reagent from tissue or cell extracts). The RNA-containing aqueous phase was separated by centrifugation, mixed with equal volume of 100% ethanol and applied to an RNeasy mini column (Qiagen). RNA was eluted in 50 ul of RNase-free water and quantified by Nanodrop 1000 (Thermo Fisher Scientific). Taqman probes against *WARS2* or *18S* were purchased from Applied Biosystems. After RNA extraction, High-Capacity RNA-to-cDNA Kit (Applied Biosystems) was used for reverse transcription reaction. Quantitative PCR process was carried using TaqMan Fast Advanced Master Mix (Applied Biosystems) on the StepOnePlus system (Applied Biosystems). The data was analysed by comparative computed tomography methods.

### Protein studies

Cells were lysed in cell lysis buffer (Cell Signalling Technology) containing protease/phosphatase inhibitor cocktail (Cell Signalling Technology) and PMSF (Cell Signalling Technology). Rat tissues were rinsed in ice-cold PBS and snap-frozen in liquid nitrogen. Tissues (∼50 mg) were homogenized (MagNA Lyser; Roche) in 1 ml of cell lysis buffer (Cell Signalling Technology) containing protease inhibitors (Roche), phosphatase inhibitors (Roche) and homogenizing beads. Supernatants were then cleared by centrifuging homogenate for 20 min at 14,000 r.p.m., 4 °C. Protein concentrations were determined using the BCA protein assay reagent (Thermo Scientific). Protein samples (16–20 μg) were separated on 10–13% SDS–PAGE, transferred to PVDF membranes,and probed with antibodies. Immunoreactive bands were visualized using pierce ECL Plus western blotting substrate (Pierce) or SuperSignal west femto maximum sensitivity substrate (Pierce). HEK293 cells expressing various plasmids were harvested in 20 mM HEPES (pH 7.4), 137 mM sodium chloride, 1.5 mM magnesium chloride, 1 mM EGTA, 10% (v/v) glycerol, 1% Triton X-100, a mixture of protease inhibitors (Roche), and 0.2 mM sodium orthovanadate. Cell lysates were precleared by centrifugation at 16,000*g* for 15 min, and supernatants were incubated with FLAG M2 agarose-conjugated beads (A2220, Sigma) for 2 h at 4 °C. Immunoprecipitates were collected by centrifugation and washed three times with lysis buffer. The resulting immunoprecipitates were separated on SDS–PAGE and immunoblotted with various primary antibodies below: rabbit anti-WARS2 (1:1,000, NBP1-54653, Novus), mouse anti-WARS2 (1:1,000, clone 2E3E8, GenScript), rabbit anti-GAPDH (1:1,000, 2118, Cell Signalling Technology), rabbit anti-FLAG (1:5,000, F7425, Sigma), rabbit anti-TOM20 (1:1,000, sc-11415, Santa Cruz), rabbit anti-COX11 (1:1,000, sc-98918, Santa Cruz) and mouse anti-β-Actin antibody (1:5,000, A5441, Sigma). All western blot data shown are representative of at least three separate individual experiments, unless otherwise stated. Full scans of western blots are available in Supplementary Fig. 16.

### WARS and WARS2 AMP-glo enzyme assay

The WARS and WARS2 enzyme assay was performed using an ATP depletion assay based on a chemilumines optimized with the AMP-GloTM reagent (Promega) using 96-well format assay. Reaction buffer contained 25 mM Tris-HCL pH7.2, 10 mM MgCl2, 50 mM KCl, 2.5 mM dithiothreitol, 0.1 mg ml^−1^ bovine serum albumin, 0.2 mM spermine, 10 U ml^−1^ pyrophosphatase and enzyme concentration used was 200 nM. Substrate concentrations were as follows: L-tryptophan 100 μM, bulk E. coli tRNA (Sigma) 200 μg ml^−1^ and ATP 100 μM. Reaction was carried out at 37 °C for 1 h, followed by adding reagent I (AMP-glo kit). The plate was incubated at room temperature for another hour. At the end of the hour the AMP detection solution was added to all the samples and incubated for one hour at room temperature. Data was collected by measuring the luminescence with the Infinite M200 microplate Reader (Tecan).

### Zebrafish studies

Zebrafish were maintained according to Institutional Animal Care and Use Committee protocols. Wild-type *AB* and *Tupfel long fin* fish were used throughout the study. In certain experiments, transgenic lines expressing GFP or dsRed under control of the vascular/endothelial growth factor receptor (*Tg(kdrl:GFP))* and (*Tg(kdrl:dsRed)*) or the cardiac myosin light chain 7 *(Tg(myl7:GFP))* promoter were used to delineate ECs and cardiomyocytes, respectively.

### Zebrafish gene knockdown and rescue

A morpholino antisense oligonucleotide (Gene Tools, LLC) was designed to knock down gene expression of *wars2* (Genbank accession number NM_001013323). The *wars2*ATG morpholino with sequence 5′-TCCACCTTATGGACAGCGCCATCTT-3′ blocks translation of *wars2* mRNA by interfering with ribosome binding to the translation start site. The morpholino was resuspended in sterile water to a concentration of 1 mM. After further dilution in Danieau's solution (58 mM NaCl, 0.7 mM KCl, 0.4 mM MgSO_4_, 0.6 mM Ca(NO_3_)_2_, 5 mM Hepes) to the desired concentration, 1 nl was injected into fertilized eggs at the single cell stage using an Eppendorff FemtoJet. After injection, the embryos were kept at 28 °C in E3 solution (5 mM NaCl, 0.17 mM KCl, 0.33 mM CaCl_2_, 0.33 mM MgSO_4_).The human wild-type *WARS2* coding sequence was cloned downstream of the upstream activating sequence (*UAS)* enhancer into the Tol2kit expression system using Gateway technology (Life Technologies). To rescue the phenotype of *wars2* knockdown, the human *WARS2* construct (20 ng μl^−1^) together with 10 ng μl^−1^ capped Tol2 transposase mRNA were coinjected with *wars2* morpholino into one-cell-stage zebrafish embryos expressing the yeast transactivator protein Gal4 under control of the ubiquitin promoter *Tg(ubi:Gal4)*.

### Zebrafish imaging

Zebrafish embryos were observed for up to 14 days after fertilization using an SZX16 stereomicroscope (Olympus). Images were recorded using an Axiocam MRc digital camera (Zeiss). Fluorescent images were obtained using an Eclipse Ti-E inverted microscope (Nikon) with a Neo 5.5 sCMOS camera (ANDOR). Time-lapse fluorescent recordings were performed using automated imaging of lightly anaesthetised (150 mg l^−1^ tricaine) zebrafish embryos embedded in 0.5% low-melt agarose in a 96-well plate, enabling simultaneous recordings of multiple embryos. Confocal images of isolated hearts were captured on a Leica SP5X laser scanning confocal microscope. Two-photon imaging of hearts and blood vessels in anaesthetised zebrafish embryos fixed in 1% agarose was performed using a Zeiss LSM710 NLO Confocal/Multiphoton upright microscope equipped with a Mai Tai Ti-Sapphire laser (Spectraphysics). Images were processed and analysed using NIS-Elements Ar (Nikon), Imaris (Bitplane) and ImageJ (NIH) software.

### Zebrafish cardiac function

For analysis of cardiac function, embryos were laterally positioned. Video microscopy was performed on an Axioplan (Zeiss) upright microscope with a FastCam-PCI high-Speed digital camera (Photron). A total of 1,000 frames were digitally captured at identical frame rates (250 frames per second) and magnification (× 5). Heart rate and cardiac output were calculated by analysis of sequential images using NIH ImageJ. Three consecutive measurements were made from each heart.

### Western blot on zebrafish samples

Whole zebrafish embryos were homogenized in RIPA buffer (50 mM Tris-HCl, 150 mM NaCl, 1% NP-40, 0.5% sodium deoxycholate, and 0.1% SDS, pH 7.4), containing protease inhibitors (Sigma). Protein samples were separated on 10% SDS–PAGE and transferred to PVDF membranes. Blots were then probed with rabbit antibodies raised against human Wars2 (1:1,000; Aviva Systems Biology) or GAPDH (1:5,000; Cell Signalling Technologies), and secondary anti-rabbit antibody coupled to horseradish peroxidase (Cell Signalling Technologies). Immunoreactive bands were detected using ECL Select Western Blotting Detection Reagent (GE Life Sciences).

### WARS2 gene targeting and studies of F1 rats

All experiments using *Wars2* targeted rats and the F1 rats derived from crossing this to the SHR were carried out at the Duke-NUS Graduate Medical School Singapore. Animals were housed at a maximum of five per cage with ad libitum access to standard rat chow and sterile water. With exceptions to breeding purposes, animals were separated according to sex. Rats were maintained on a twelve-hour diurnal cycles by automatic light switching. All procedures were performed in accordance with local IACUC approvals granted by National University of Singapore. Male rats aged 10–16 weeks were used for all studies.

### Disruption of Wars2 in the rat

The BN rat *Wars2* locus (wild-type allele) was targeted in the terminal region of the first exon with ZFN against the ZFN Target site: ACCCACAGCTACTGCggctcCCCAGGTAACCCGAG (Sage laboratories, PA, USA). Gene disruption was screened by sequencing and an 8 bp deletion identified in exon 1 at the ZFN target site. This resulted in a frame shift after amino acid 27 of Wars2 protein and a premature stop in exon 2 after amino acid 53 of Wars2.

### Genotyping of mutant strains

Genomic DNA was extracted from toe clip samples using E.Z.N.A Tissue DNA kit (OMEGA Bio-Tek) according to manufacturer's protocol. Following purification, PCR was performed on each sample consisting of JumpStart Taq ReadyMix (Sigma) and 1 μM of the following primers: (i) 5′-GTGAGTGCTGGCGCTTCATC-3′ and (ii) 5′-GGCCTAAAGCAGAAGGTCGG-3′. PCR cycling conditions: (i) 95 °C for 5 min; (ii) 95 °C for 30 s; (iii) 65 °C for 30 s; (iv) 72 °C for 30 s; (v) repeat cycles 2 to 4 for 30 times; (vi) 72 C for 5 min; (vii) 4 °C hold. PCR products were run on a 5% agarose gel. Expected band sizes were (i) wild-type=92 bp (ii) heterozygous deletion=92 bp and 84 bp (ii) homozygous deletion=84 bp.

### F1 rats derived from BN (Wars2^+/−^) x SHR

BN rats carrying *Wars2*^−/+^ were backcrossed to wild-type BN rats (*Wars2*^+/+^) to produce F1 BN (*Wars2*^−/+^) rats for intercrossing. BN (Wars2^+/−^) F1 female rats were crossed with male SHR that are homozygous for the Wars2(L53F) mutation to produce a F_1_ population: F1(*Wars2*^+/L53F^) or F1(*Wars2*^−/L53F^).

### CF measurements in F1 animals

Rats were anaesthetised with Ketamine (80 mg kg^−1^) and Xylazine (10 mg/kg) cocktail via intraperitoneal injection. Heparin (1,000U) was administered subcutaneously. Experimetns were performed largely as described above for the mapping strains with the modification that the perfusion pressure was set to 70 mm Hg, which was better tolerated in F1 animals. CF was measured at baseline for 15 min and indexed to heart weight. Data were acquired and analysed using LabchartPro software (ADInstruments).

### Echocardiography

Rats were anaesthetised with 3% isoflurane during induction and were maintained at 1.6 to 2.0% isoflurane during images acquisition. Heart rates were maintained at the average of 360 b.p.m. Echocardiograms were performed on Vevo 2100 system (VisualSonics) with a linear array transducer (MS250 13–24 MHz, VisualSonics). An average of 10 cardiac cycles were stored in cine loops for subsequent offline analysis using the same system.

### RNA sequencing studies

Human heart LV tissues (*n*=128) from the Royal Brompton and Harefield Trusts transplant programme were collected with ethical approval and prepared for RNA Sequencing studies as previously described^54^. After sequencing, reads were de-multiplexed and mapped to the human genome (GRCh37) and transcriptome using TopHat 1.4.1. Read numbers were quantile-normalized for correlation analyses of *WARS2* expression. RNA Seq data have been deposited in EMBL-EBI under accession code: PRJEB8360 (http://www.ebi.ac.uk/ena/data/view/PRJEB8360 (data available online).

### Statistical analyses

Statisitcal analyses were carried out in R, Matlab or GraphPad Prism. Comparisons between groups were performed using Mann-Whitney, Student's *t*-Test or analysis of variance as appropriate. CF QTLs were mapped using a Matlab implementation of ESS^17^. To compute robust pairwise correlations between *ARS2* genes and others genes, Tukey's biweight method was used as implemented in WGCNA package for R.

### Data availability

The RNA sequence data were deposited at EMBL-EBL under Accession code: PRJEB8360. All other relevant data are available from the authors on request.

## Additional information

**How to cite this article**: Wang, M. *et al*. *Wars2* is a novel determinant of angiogenesis. *Nat. Commun.* 7:12061 doi: 10.1038/ncomms12061 (2016).

## Supplementary Material

Supplementary Information Supplementary Figures 1-18 and Supplementary Table 1

Supplementary Data 1 Wars2(L53F) mutation across inbred rat strains

Supplementary Data 2 Top 20 transcripts most significantly correlated with *WARS2* expression (top 10 +ve; top 10 -ve)

Supplementary Movie 1 control fish vessel detail

Supplementary Movie 2 control fish whole embryo

Supplementary Movie 3 wars2 MO(0.5ng) fish vessel detail

Supplementary Movie 4 wars2 MO(0.5ng) fish whole embryo

Supplementary Movie 5 wars2 MO(1.0ng) fish vessel detail

Supplementary Movie 6 wars2 MO(1.0ng) fish whole embryo

Supplementary Movie 7 control fish heart

Supplementary Movie 8 wars2 MO(1ng) fish heart
