# Supplementary material for: Wars2 is a determinant of angiogenesis
Source: Nat Commun. 2016 Jul 8;7:12061. doi: 10.1038/ncomms12061 (PMC4941120; doi:10.1038/ncomms12061)
Supplement: Supplementary Information — Supplementary Figures 1-18 and Supplementary Table 1 [file ncomms12061-s1.pdf]

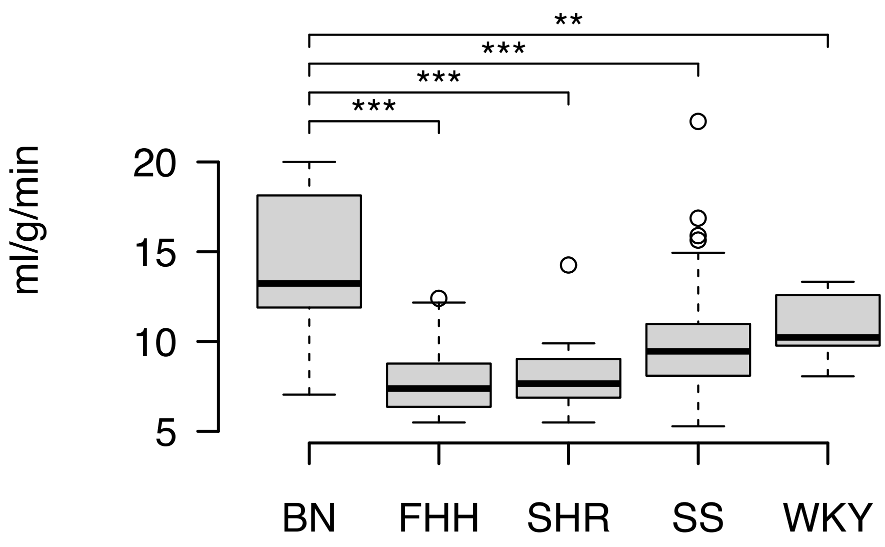

Supplementary Fig. 1. The Brown Norway rat has higher coronary flow compared to other rat strains.
Publically available data for coronary flow measured ex vivo on Langendorff apparatus under intrinsic heart rate conditions were retrieved from http://pga.mcw.edu/ and plotted for a number of rat strains relevant to our studies: Brown Norway (BN), Fawn Hooded Hypertensive (FHH), Spontaneously Hypertensive (SHR), Salt Sensitive (SS) and Wistar Kyoto (WKY). n>10 per genotype; see also at http://pga.mcw.edu/. ANOVA with post-hoc Tukey: **, P<0.01; ***, P<0.001.

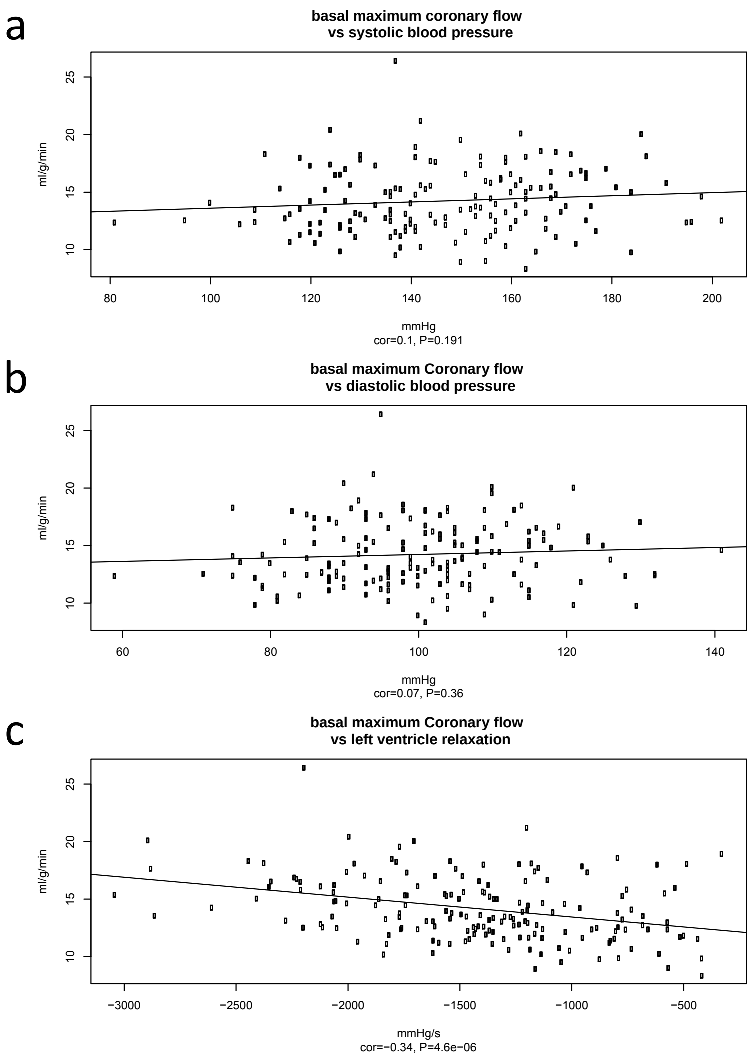

Supplementary Fig. 2. Correlation of maximal coronary flow (CF) with systolic blood pressure (a), diastolic blood pressure (b) and left ventricular relaxation (c) across the F2 rat population (n=172). CF indexed to heart weight is shown on the Y axis. X-axes: a, systolic blood pressure (mmHg); b, diastolic blood pressure (mmHg); c) rate of change of LV pressure during diastole (mmHg/s).

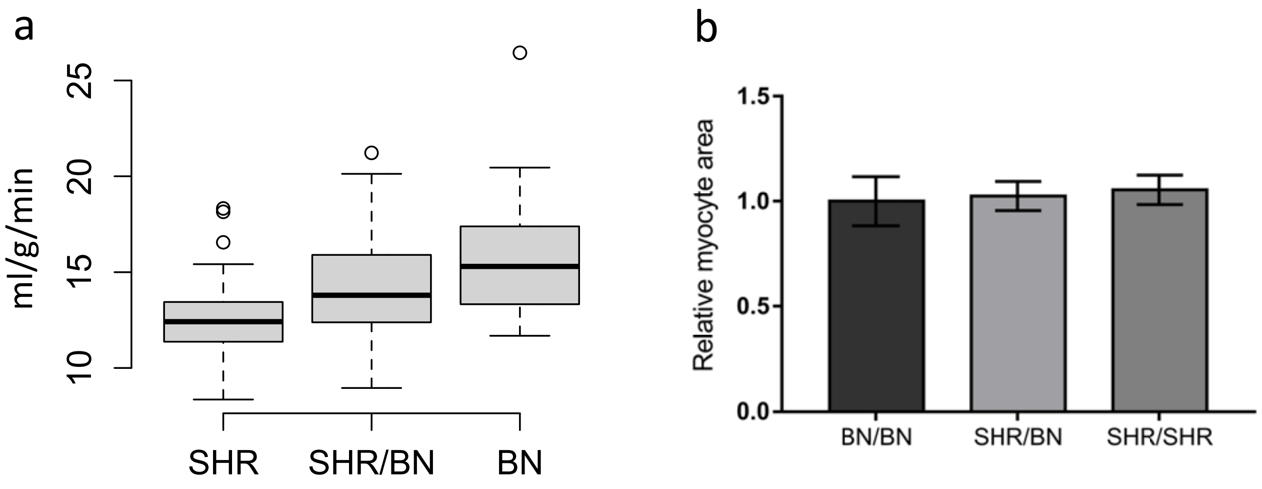

Supplementary Fig. 3. Coronary flow and myocyte area by parental rat genotype at the 2q34 locus (peak SNP, Chr 2: 191,677,064 bp) in the F2 population. SHR, homozygous for SHR genotype; SHR/BN, heterozygous for SHR and BN genotypes; BN, homozygous for BN genotype (n=172 in total, n >30 per genotype). b, n=7, one-way ANOVA, no significant difference.

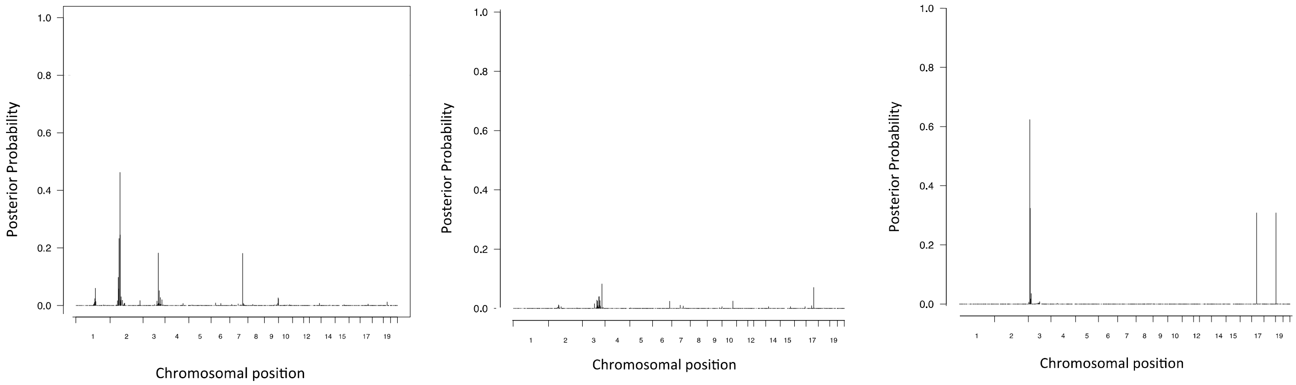

Supplementary Fig. 4. Mapping of blood pressure indices and of heart weight indexed to body weight to the genome using in the F2 intercross (n=172). Rat autosomes are listed on the x-axis and the y-axis shows the posterior probability for an association at a locus. None of these indices mapped to the CF locus on chromosome 2.

Ctss mutation in SHR rat

Chr2:190456450, G>T position 751 of the transcript ENSRNOT00000028732 (ensembl version 59): glycine > cysteine at amino acid 251 of rat Ctss.

```
BN Rat   -KEAVATKGPVSVGIDDASHSSFFLYQSGVY-
SHR RAT  -KEAVATKGPVSVCIDDASHSSFFLYQSGVY-
Human    -KEAVANKGPVSVGVD-ARHPSFFLYRSGVY-
Mouse    -KEAVATKGPVSVGID-ASHSSFFFYKSGVY-
Dog      -KEAVANKGPVSVAID-ASHYSFFLYRSGVY-
Chicken  -KDAVANVGPVSVAID-ATQPTFFLYRSGVY-
ZFish    -KQAVASVGPISVAID-ATRPQFVLYHSGVY-
```

Supplementary Fig. 5. Protein variation in SHR rat Ctss gene. There is a G>T variant at position 751 of the rat *Ctss* transcript which is predicted to be damaging and is conserved between rat, human and mouse but not between rat, dog, chicken or zebrafish (ZFish). The Ctss gene, is expressed most highly in B cells and monocytes: see also at http://www.humanproteomemap.org/protein.php?hpm_id=1520.

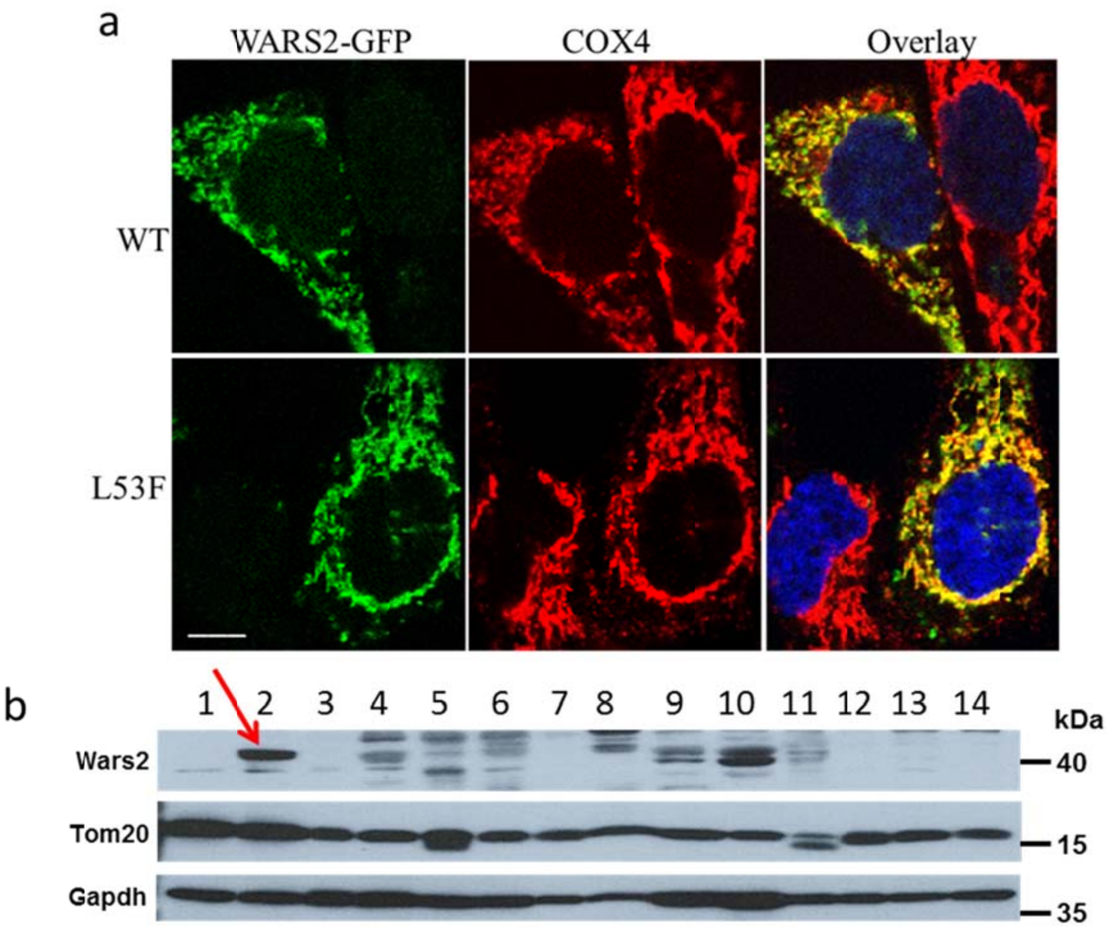

Supplementary Fig. 6. Sub-cellular localization of wild type and L53F mutant WARS2 and expression of Wars2 protein across rat tissues. a) Confocal microscopy localization of wild type (WT) WARS2 and L53F mutant WARS2 (L53F) to the mitochondria. HUVEC cells were transfected with either WT *WARS2* or *WARS2(L53F)* conjugated to GFP. Scale bar=20μm. Colocalisation of WT and L53F WARS2 to the mitochondria was shown by counter-staining with the mitochondrial protein COX4. b) Immunoblot of Wars2 and Tom20 (mitochondria) expression in cell lines (lanes 1 and 2) and across rat tissues (lanes 3-14). 1, HEK cells + vector; 2, HEK cells + Flag-WARS2 (red arrow); 3, brain; 4, heart; 5, kidney; 6, liver; 7, lung; 8, pancreas; 9, soleus muscle; 10, quadriceps muscle; 11, intestine; 12, spleen; 13, testis; 14, ovary. And see http://www.humanproteomemap.org/protein.php?hpm_id=10352.

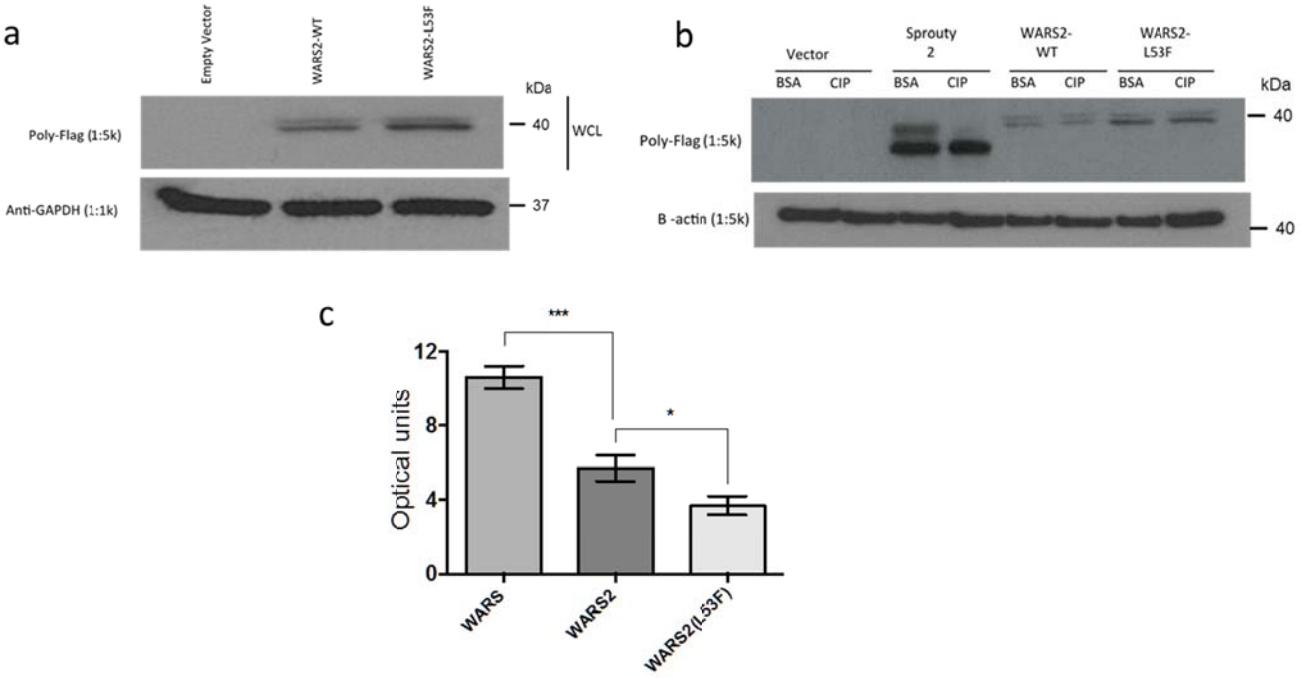

Supplementary Fig. 7. WARS2 and WARS2(L53F) protein isoforms and WARS, WARS2 and WARS2(L53F) enzyme activity (c). a and b, WARS2(L53F) mutant protein consistently exhibits a greater proportion of the faster migrating band (a), which can be observed following a dephosphorylation event. Incubation of WARS2 wild type (WT) or L53F mutant (both Flag tagged) with calf-intestinal alkaline phosphatase (CIP) did not induce a band shift as compared to incubation with bovine serum albumin (BSA). In contrast phosphorylated FLAG-Spry2 protein shows a clear band shift following dephosphorylation by CIP. Hence the WARS2 isoform with the slower migration does not represent a phosphorylation event. The lower isoform was found in greater abundance in the mitochondria (data not shown). c, In vitro enzyme assay of canonical ARS enzyme activity for WARS, WARS2 and WARS2(L53F). n=3, one-way ANOVA with Tukey's multiple comparisons test. ***, $P < 0.001$; *, $P < 0.05$.

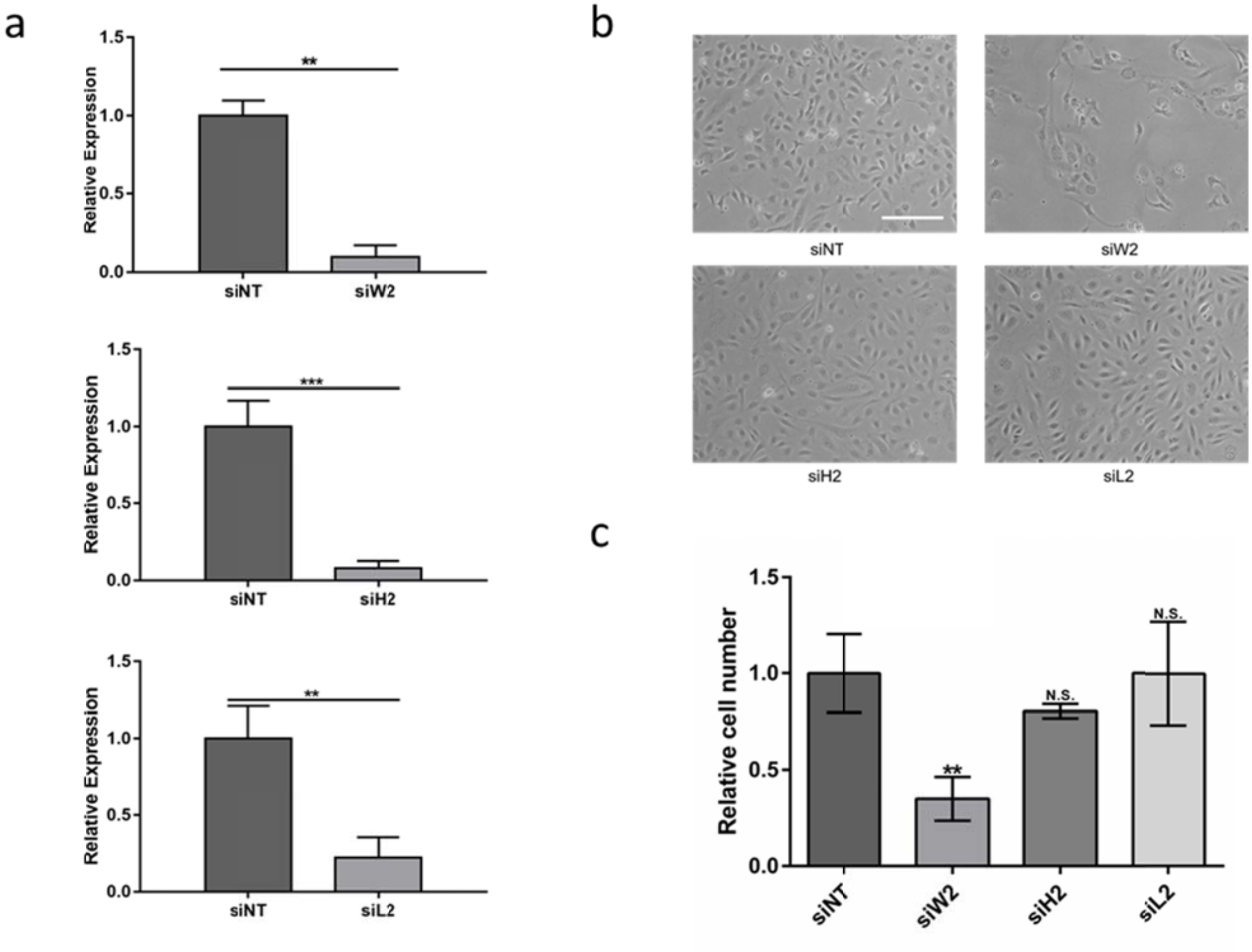

Supplementary Fig. 8. Expression of *WARS2*, *HARS2* and *LARS2,* cell morphology and number after gene silencing using siRNAs in endothelial cells. a, Relative *WARS2*, *HARS2* and *LARS2* gene expression in endothelial cells (ECs) after siWARS2 (siW2), siHARS2 (siH2), siLARS2 or siNon-targeting (siNT) (n=3 per condition, *t*-test). b, EC morphology after siNT, siW2, siH2 and siL2 treatment. Scale bar = 200 µm. c, Relative cell number after siNT, siH2 and siL2 treatment. n=3, one-way ANOVA with Tukey's multiple comparisons test. **, P<0.01; ***, P<0.001

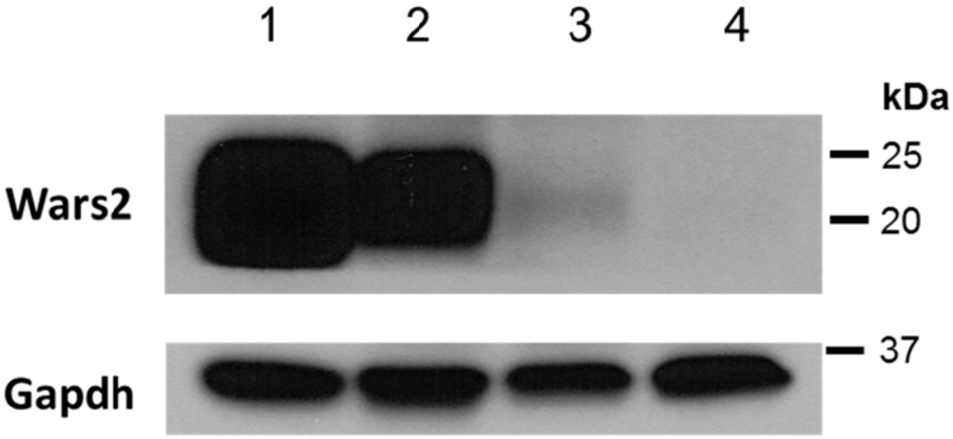

Supplementary Fig. 9. Expression of *wars2* in zebrafish and its knockdown by morpholinos.
Western blot of 5dpf zebrafish whole body lysates in control samples (1) and in fish with morpholino-mediated *wars2* knockdown (lane 2: 0.25 ng morpholino, lane 3: 0.5 ng morpholino, and lane 4: 1 ng morpholino).

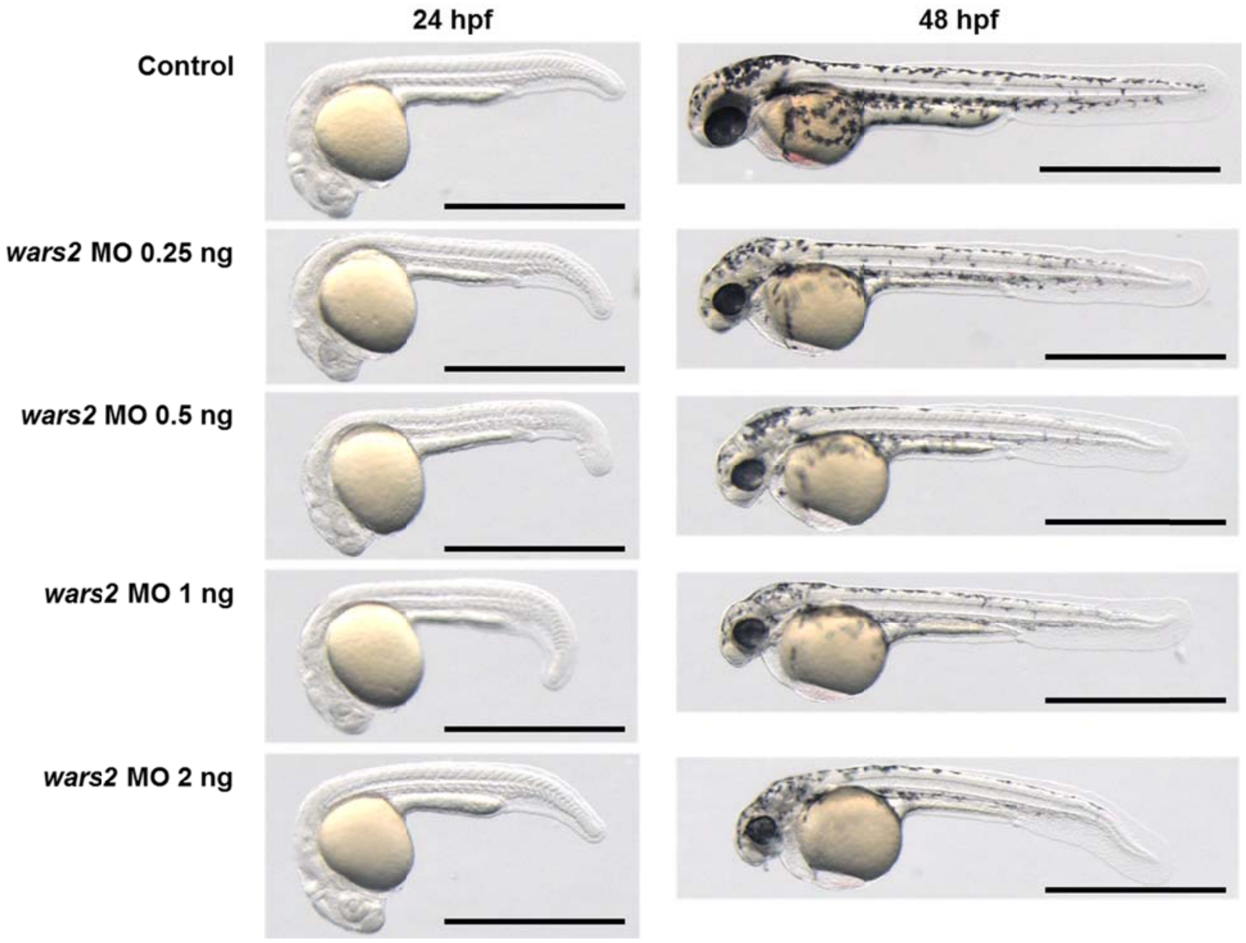

Supplementary Fig. 10. Gross phenotype of *wars2* MO injected embryos at 24 and 48 hours post fertilization. *wars2* knockdown has very little effect on gross morphology at 24 hpf, but at 48 hpf gross defects including cardiac edema and a curved body axis become evident, dependent on the *wars2* MO dose. Scale bar=1 mm.

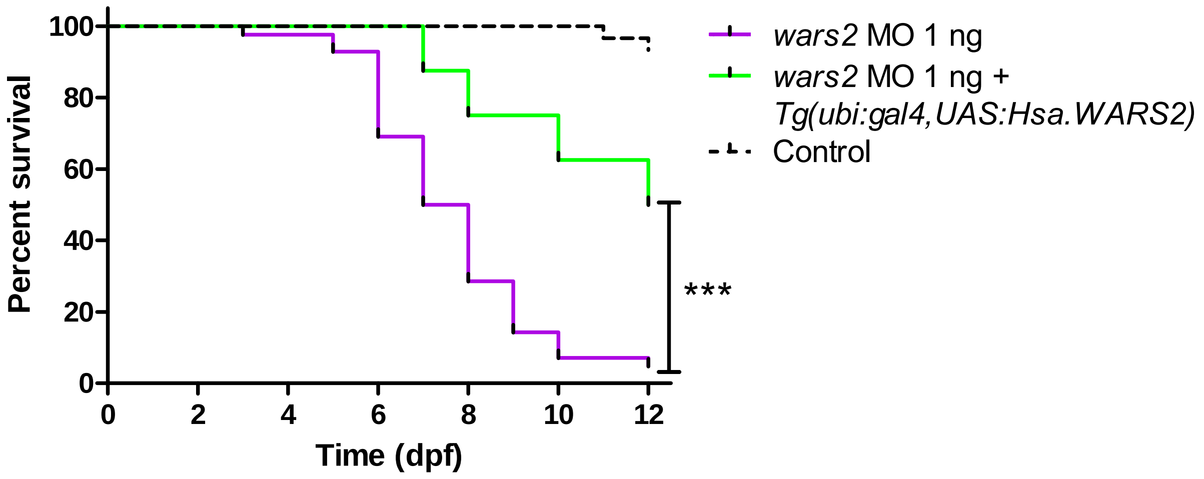

Supplementary Fig. 11. Expression of transgenic *WARS2* rescues death of zebrafish with knockdown of *wars2*. Survival of zebrafish embryos expressing the *Gal4* transcription activator under control of the ubiquitin (*ubi*) promoter injected with *wars2* morpholino (1 ng) with or without a transgenic construct encoding human *WARS2* under control of the upstream activating sequence (*UAS*) enhancer. Only zebrafish with strong expression of the *α-crystallin:YFP* marker (indicating successful incorporation of the *WARS2* transgene) and green fluorescent hearts (marker for the *ubi:Gal4* genotype) were included for analysis in the *wars2* MO 1 ng + *Tg(ubi:gal4, UAS:Has.WARS2)* group. N=42 for *wars2* MO 1ng, 8 for *wars2* MO 1ng + *Tg(ubi:gal4m UAS:Has.WARS2)*, and 30 for the Control group. ***: P<0.0001 by Log-Rank (Mantel-Cox) test.

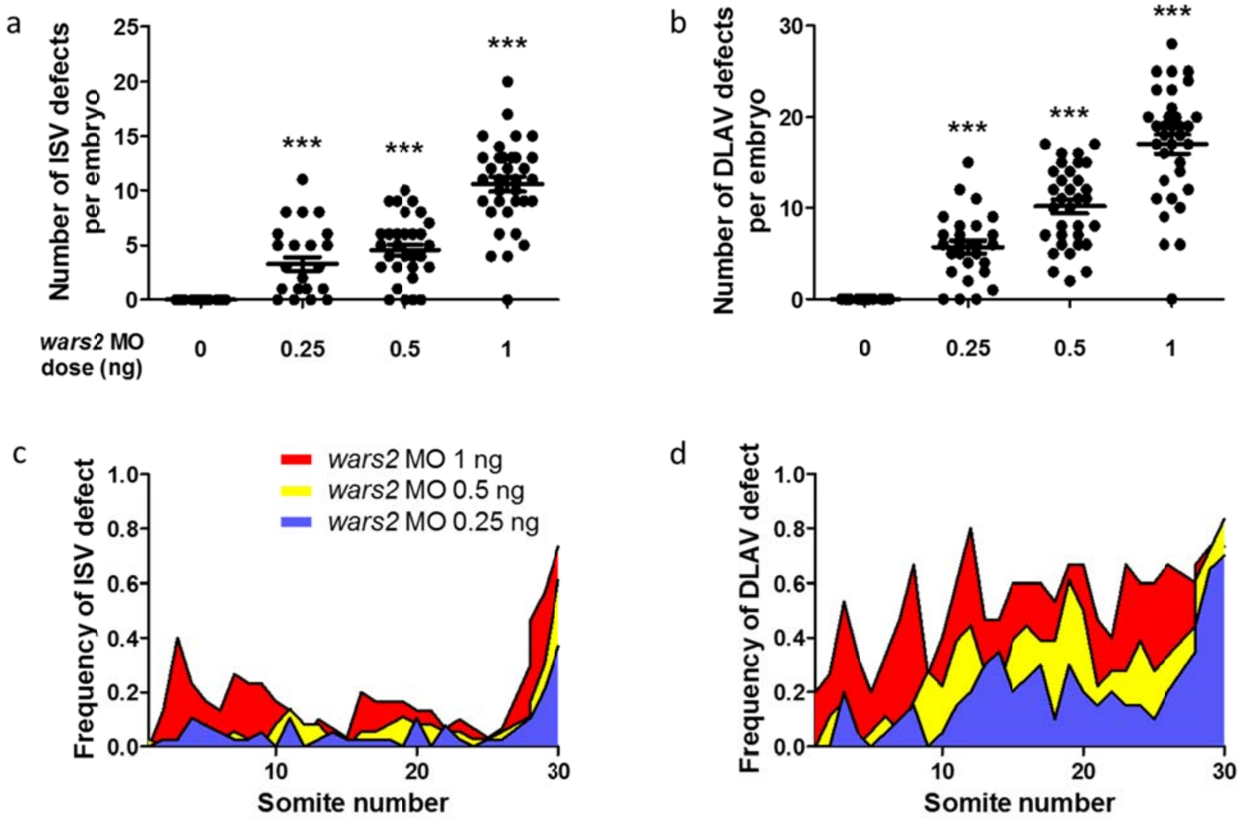

Supplementary Fig. 12. Prevalence and spatial distribution of vascular defects in embryos injected with different doses of the *wars2* morpholino. The vasculature of *Tg(flk:GFP)* embryos was systematically scored for vascular defects at 48 hpf, and the location was noted as the number of the somite (counting from the head) where the defect was observed. a-b: ***: P<0.001 vs control by Bonferroni post-test after one-way ANOVA. N=27-35 embryos per group. c-d: N=15-19 embryos per group.

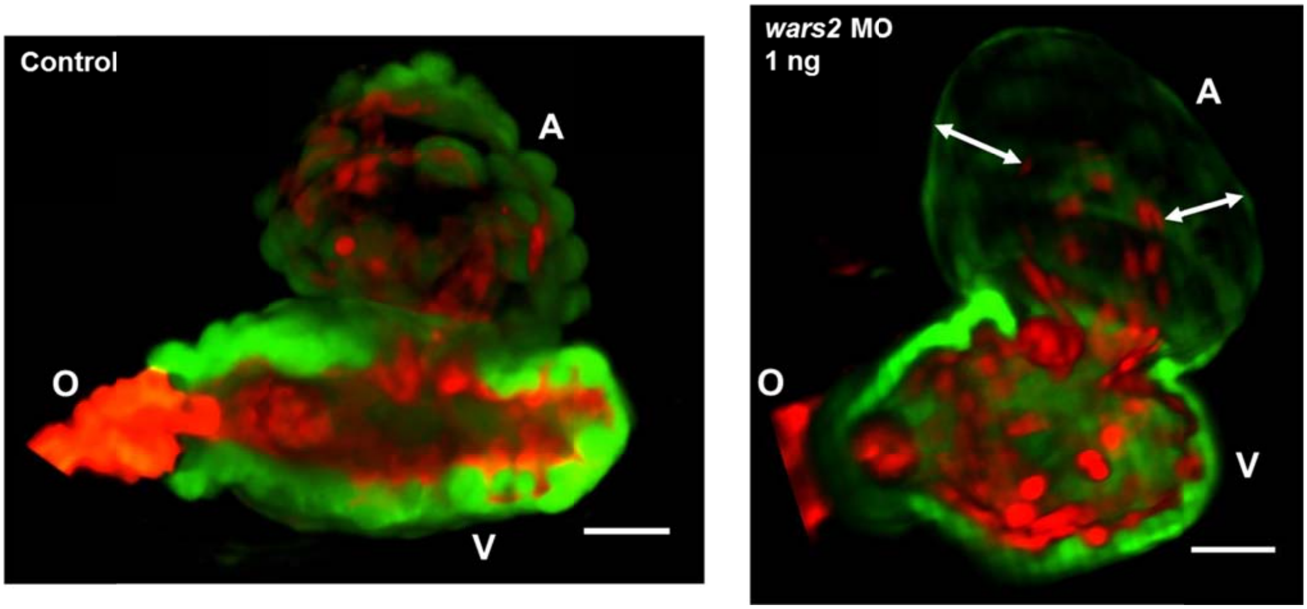

Supplementary Fig. 13. Optical slices of hearts from 4dpf *Tg(myl7:GFP;flk:dsRed)* zebrafish embryos.

Embryos challenged with 1 ng of the *wars2* morpholino showed a clear separation (arrows) of the endocardium (red) and myocardium (green). A: atrium, O: outflow tract, V: ventricle. Scale bar=30μm.

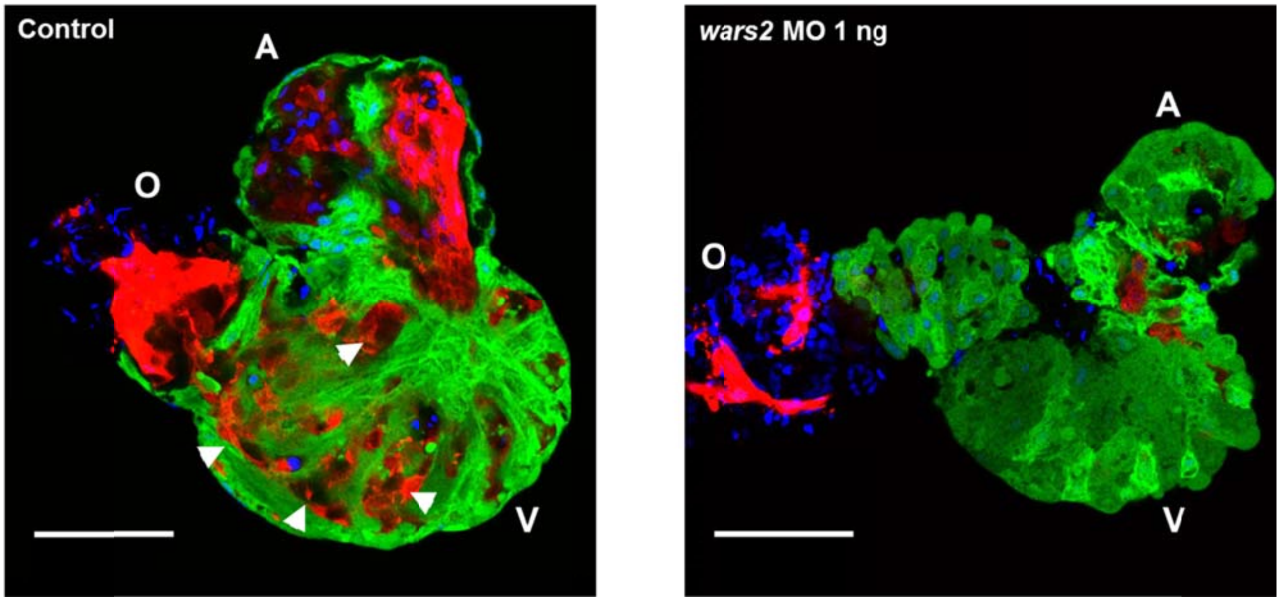

Supplementary Fig. 14. Confocal images optically sectioned at a depth of 2 µm into the wall of isolated hearts of 4dpf Tg(myl7:GFP;flk:dsRed) zebrafish embryos. In control embryos there are numerous infiltrations of endothelial cells (red) interspersed between myocardial (green) trabeculae (arrowheads), which are almost completely absent in hearts lacking Wars2. Blue: Hoechst staining. A: atrium, O: outflow tract, V: ventricle. Scale bar=50µm.

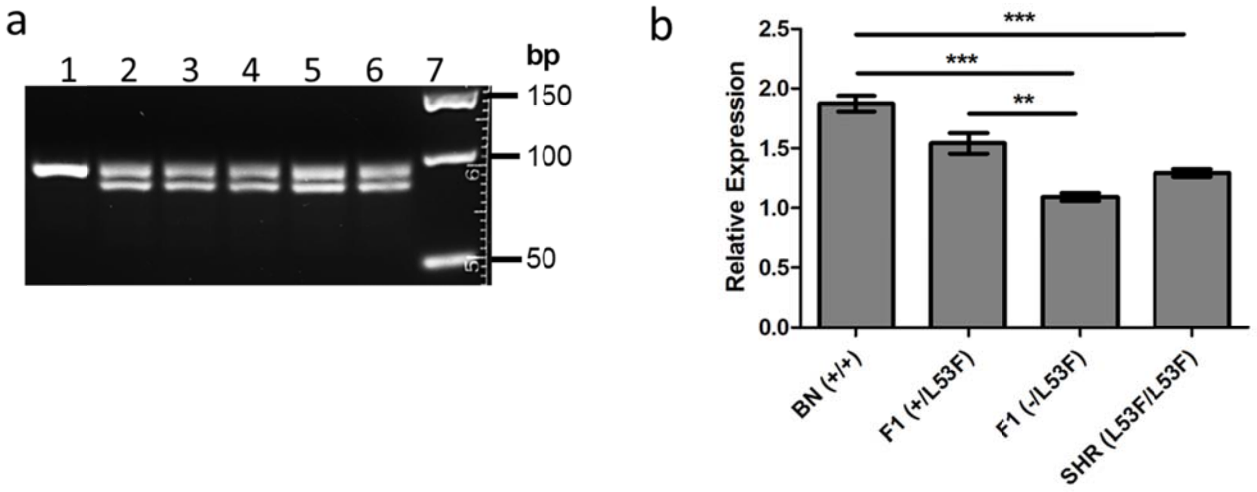

Supplementary Fig. 15. Genotyping of *Wars2* in germline targeted rats and *Wars2* expression levels by genotype. (a) Genotyping of the 8bp deletion in wild type BN(*Wars2*$^{+/+}$) and heterozygous gene targeted BN(*Wars2*$^{-/+}$) rats. Lane 1, homozygous wildtype *Wars2*$^{+/+}$; lanes 2-6, heterozygous *Wars2*$^{-/+}$; Lane 7, marker (50bp, 100bp and 150bp). (b) gene expression of *Wars2* in the heart of BN(*Wars2*$^{+/+}$) , F1(*Wars2*$^{-/+}$), F1(*Wars2*$^{-/L53F}$) and SHR (*Wars2*$^{L53F/L53F}$) rats. n=3, one-way ANOVA with Tukey's multiple comparisons test. **, P<0.01; ***, P<0.001.

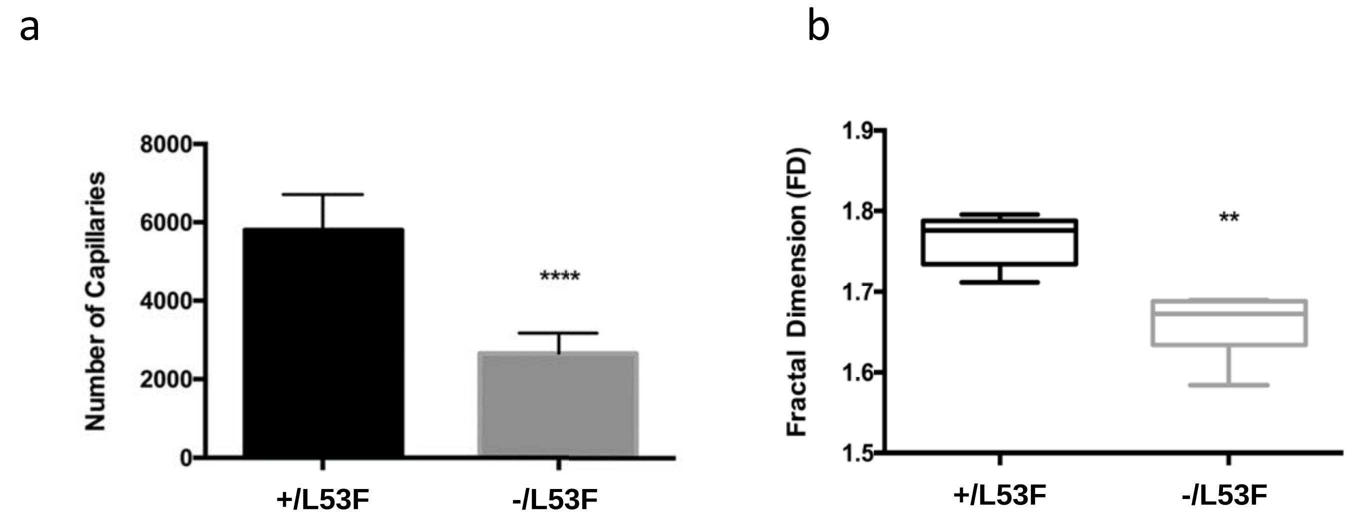

Supplementary Fig. 16. Inhibition of *Wars2* causes diminished capillary density and complexity in the rat. a, Capillary density on whole mount short axis section (showed in number of capillaries per mm^2) in F1 rats (n=5/genotype). b, Capillary complexity (quantified by fractal dimension analysis) in F1 rats (n=5 rats/genotype). t-test. **, P<0.01; ****, P<0.0001;

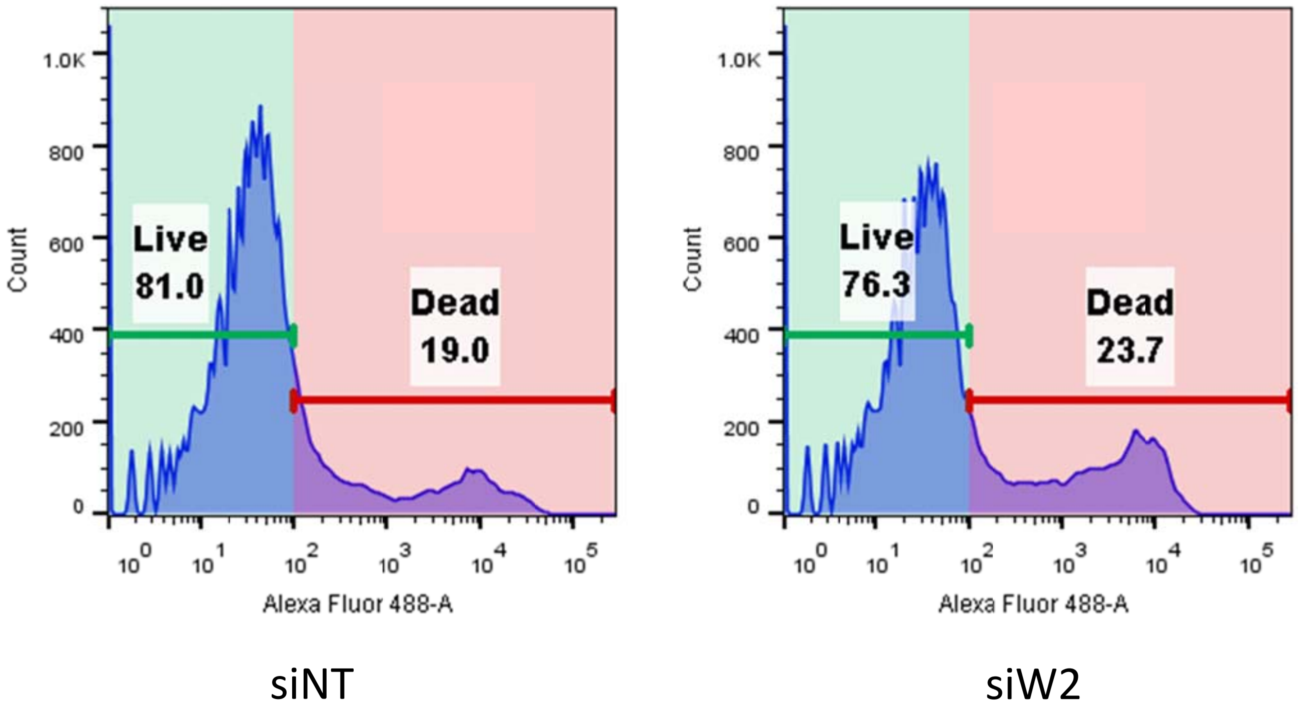

Supplementary Fig. 17. Live dead assay in HUVEC. HUVEC cells were treated with siRNA that was non targetting (NT) or against WARS2 (siW2) and cells were harvested 72 h post transfection for flow cytometry analysis by labeling with Image-iT DEAD Green viability stain. With knock-down of *WARS2* there was a right shift indicating a higher percentage of dead cells in accordance with the data using a different assay of cell death (main text, Fig. 4). The experiment was repeated with similar results.

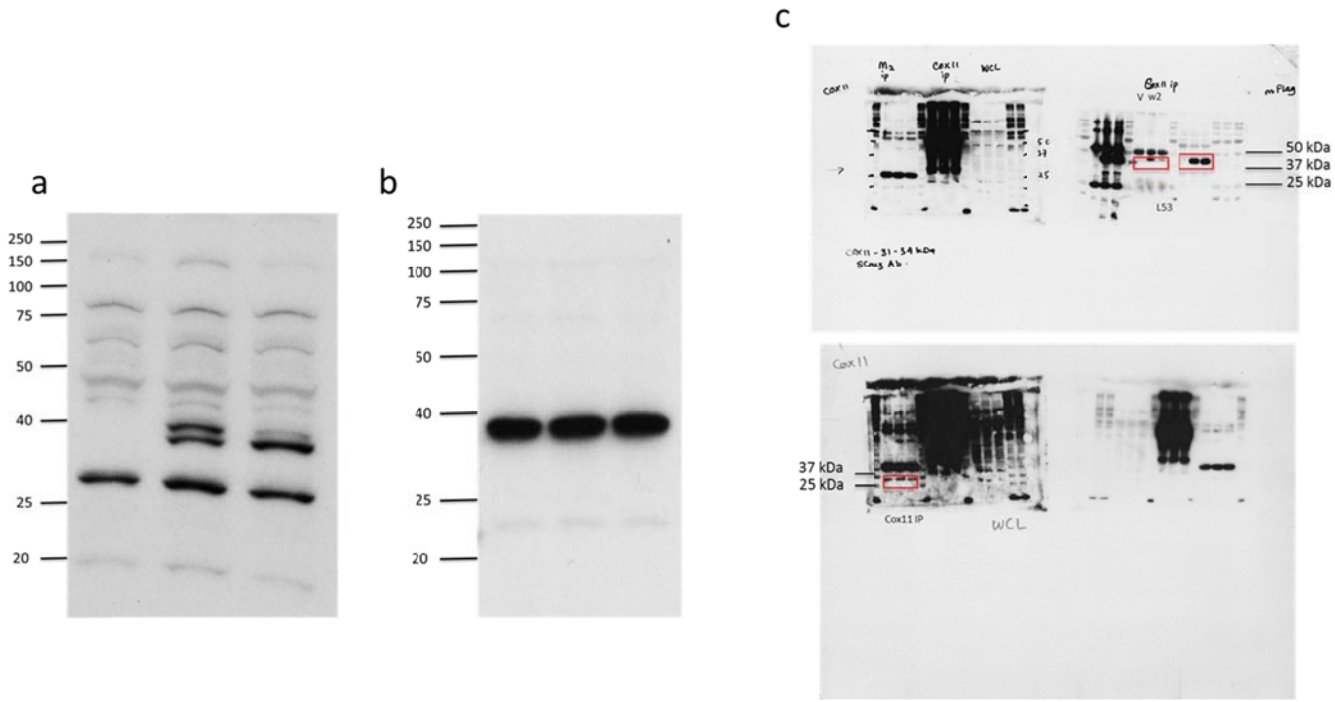

Supplementary Fig. 18. Original uncropped blots. a, Figure 1i (WARS2). b, Figure 1i (GAPDH). c, Figure 4d. Top left, FLAG-WARS2, IP: Cox11; top right, FLAG-WARS2, WCL; bottom, Cox11, IP: Cox11.

	+/L53F (n=7)	-/L53F (n=13)	*p*-value
EF (%)	76.3 ± 5.5	75.6 ± 6.8	0.8212
LVIDed (mm)	6.7 ± 0.6	7 ± 0.4	0.2136
LVIDes (mm)	3.3 ± 0.6	3.4 ± 0.7	0.5451
AWTed (mm)	2 ± 0.2	2.1 ± 0.3	0.1222
AWTes (mm)	3.1 ± 0.2	3.4 ± 0.3	0.0932
PLVWed (mm)	1.8 ± 0.1	1.8 ± 0.2	0.9254
PLVWes (mm)	3.0 ± 0.2	3.1 ± 0.2	0.1826

Supplementary Table 1. Echocardiographic data in F1(*Wars2*(+/L53F)) and F1(*Wars2*(-/L53F)) rats. Left ventricular internal diameter, LVID; anterior wall thickness, AWT; posterior left ventricular wall thickness, PLVW; end diastole, ed; end systole, es.
